# Supplementary figures and images for: Whole blood transcriptome profile identifies motor neurone disease RNA biomarker signatures
Source: Exp Biol Med (Maywood). 2025 Jan 8;249:10401. doi: 10.3389/ebm.2024.10401 (PMC11750576; doi:10.3389/ebm.2024.10401)

A

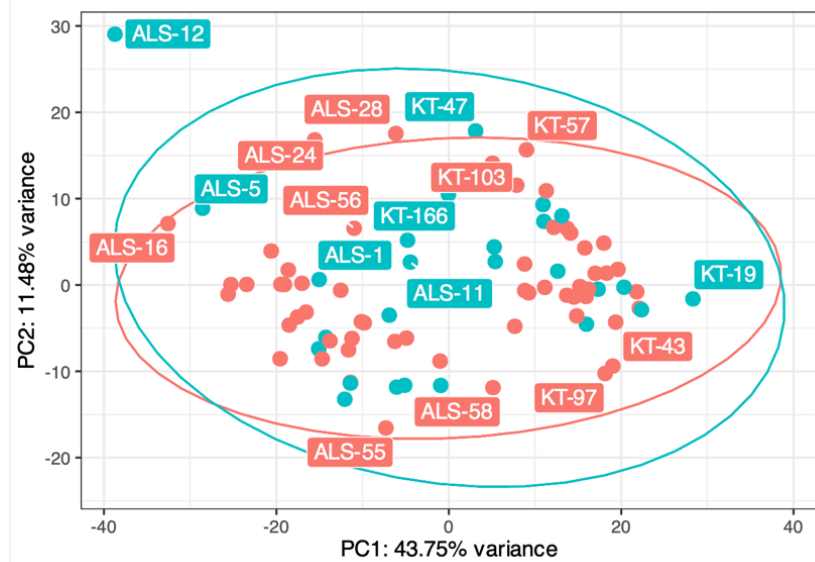

B

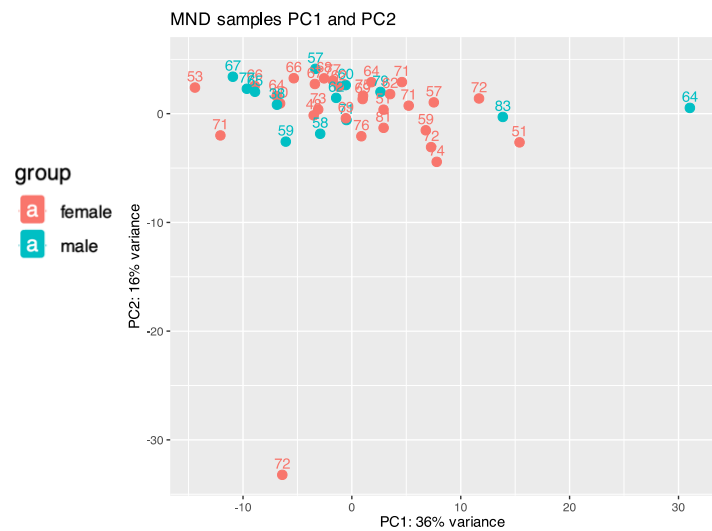

C

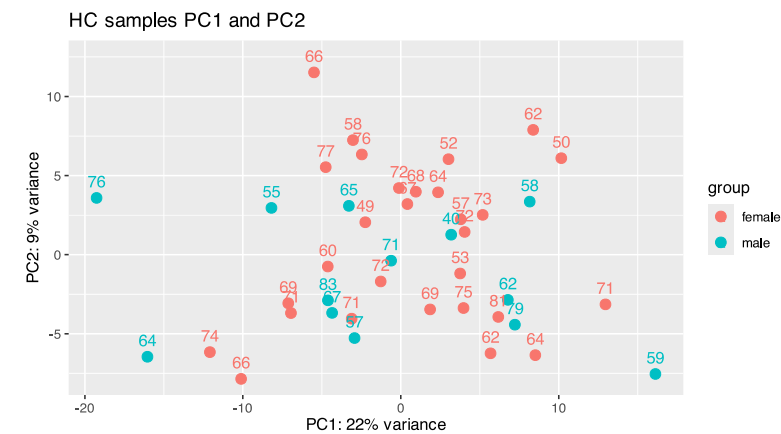

D

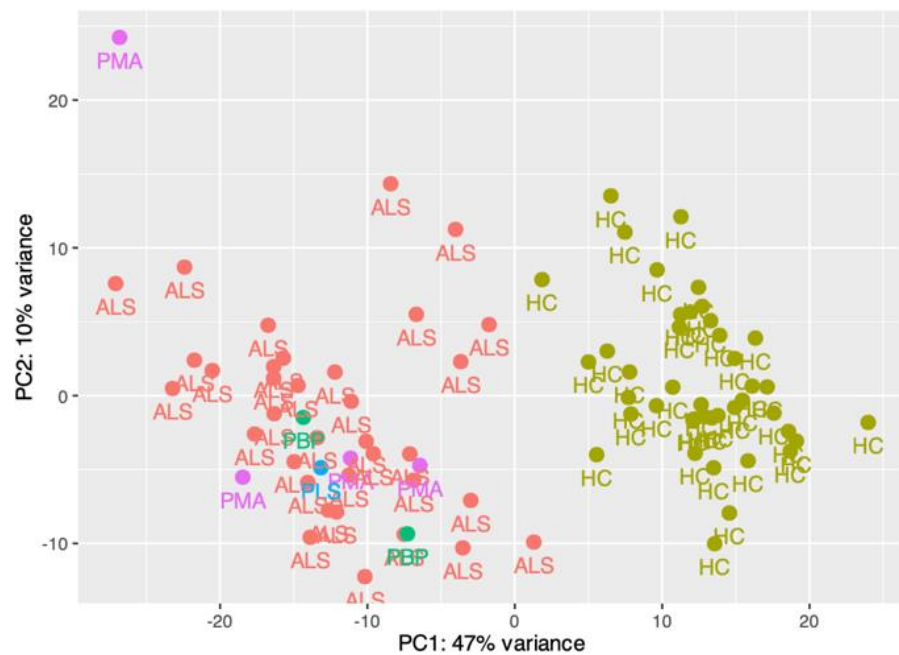

E

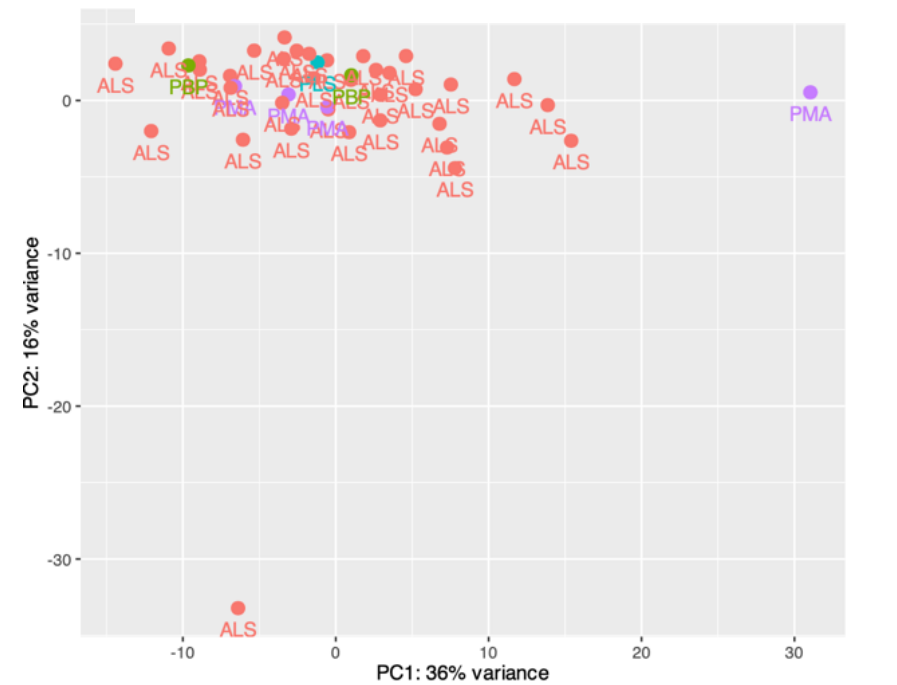

Supplement: Supplementary file 3 [file Image2.PDF]

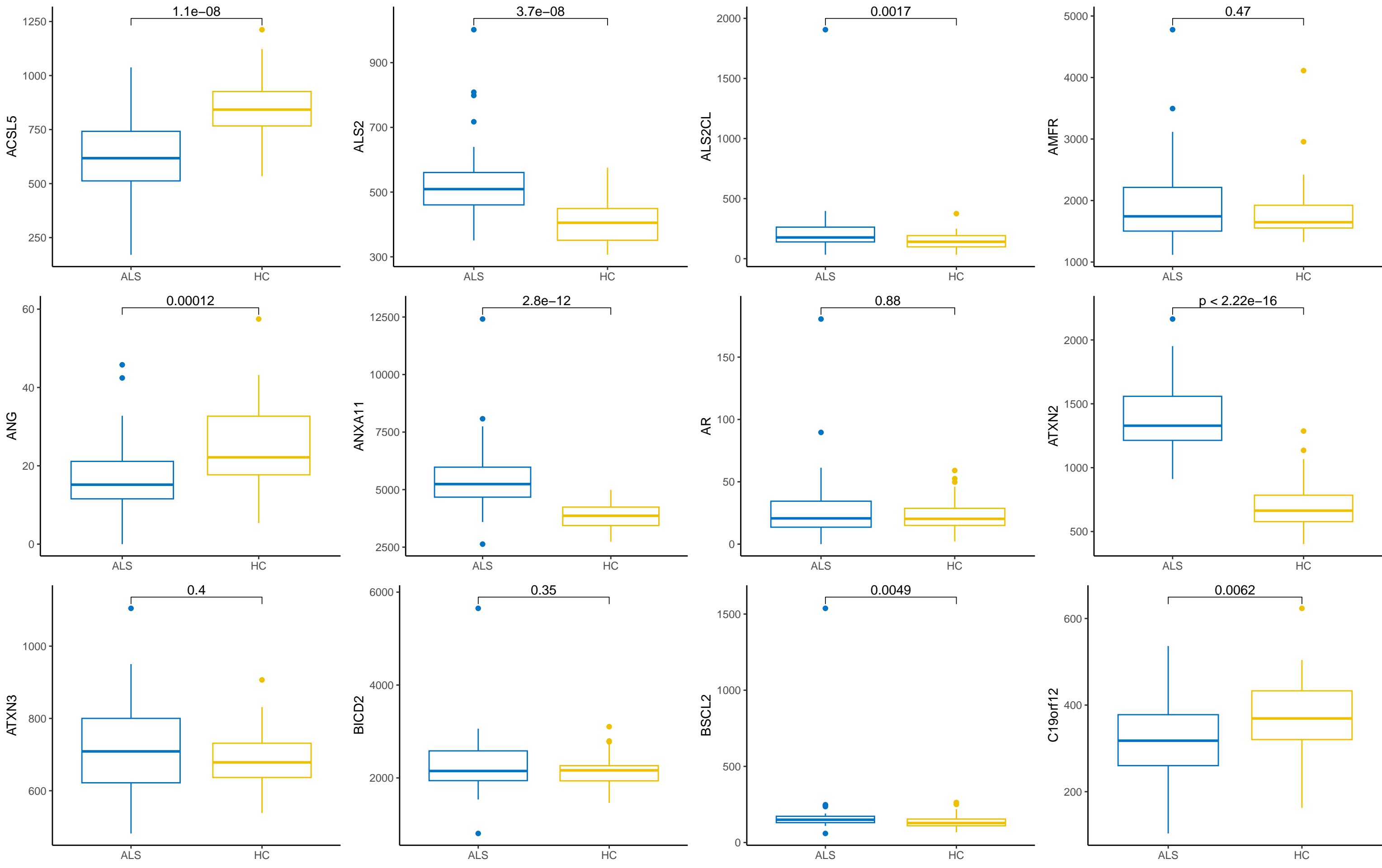

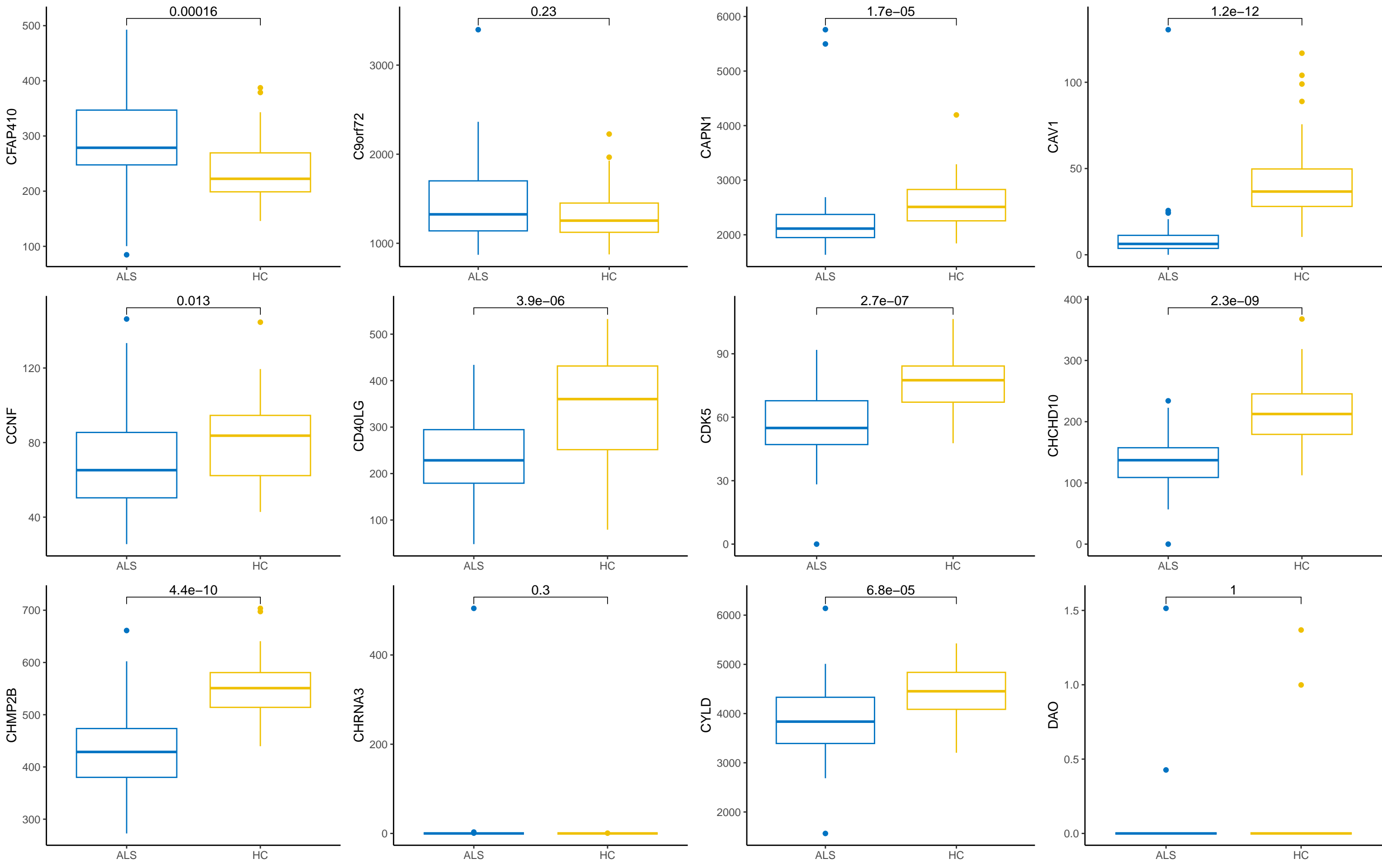

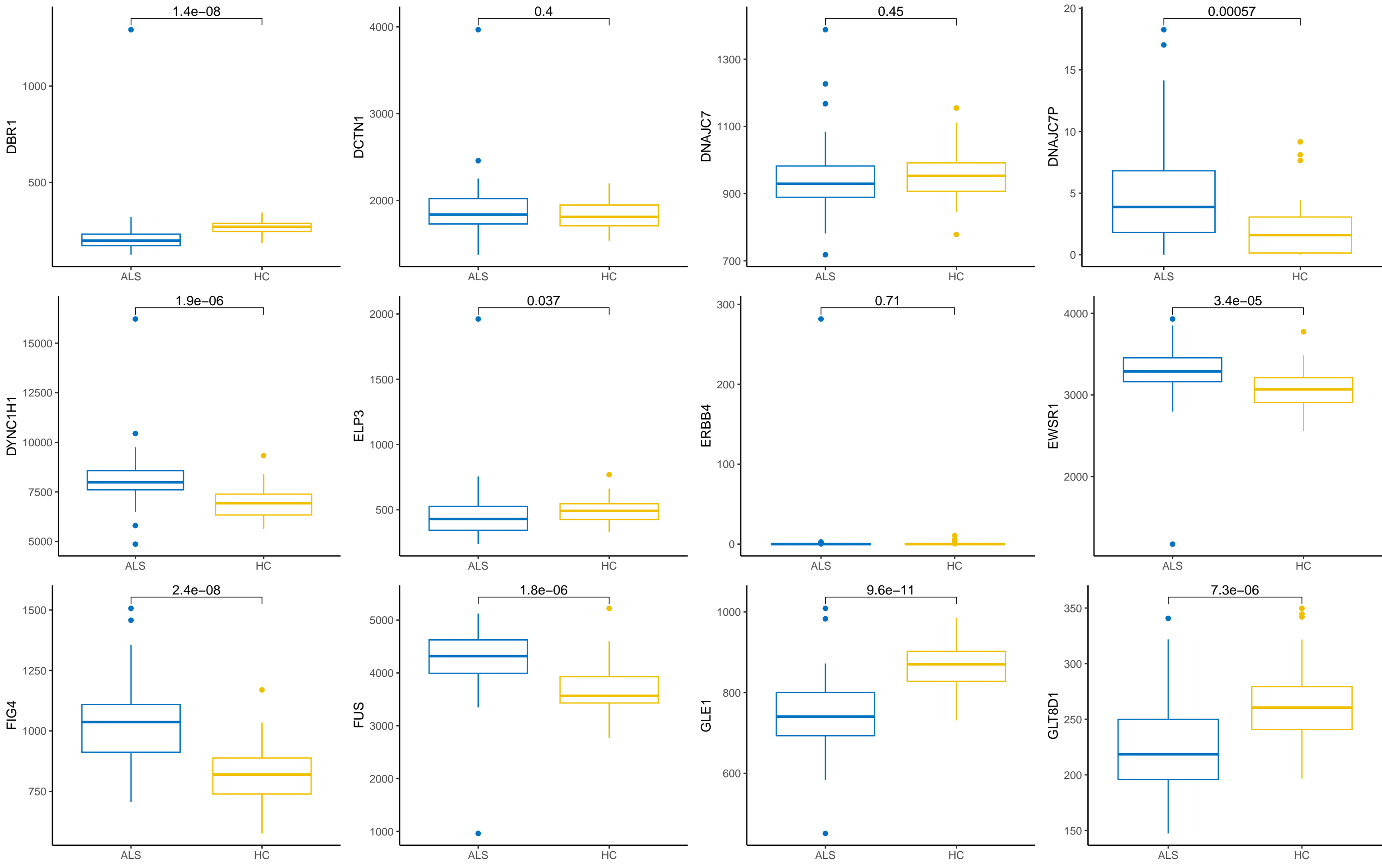

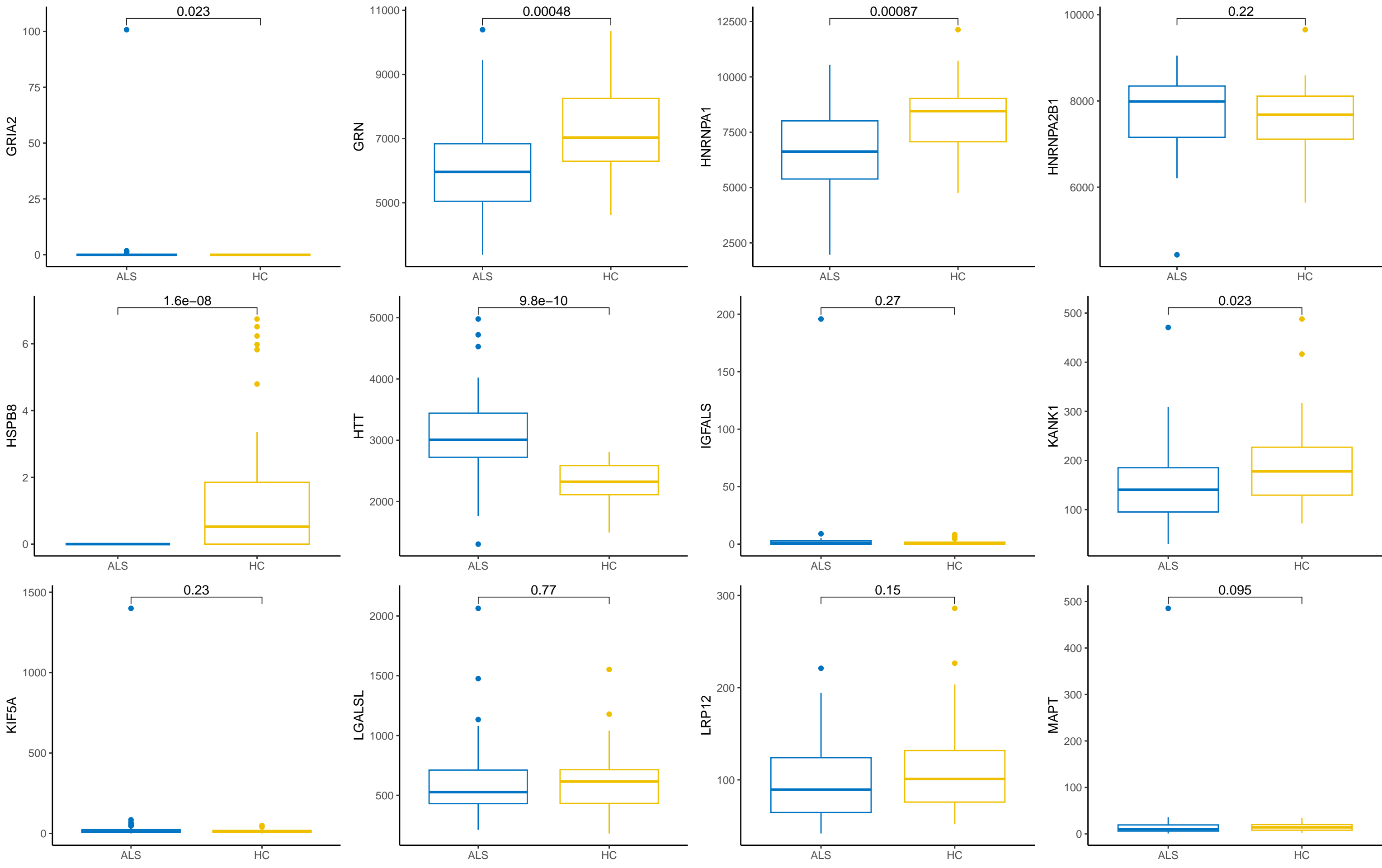

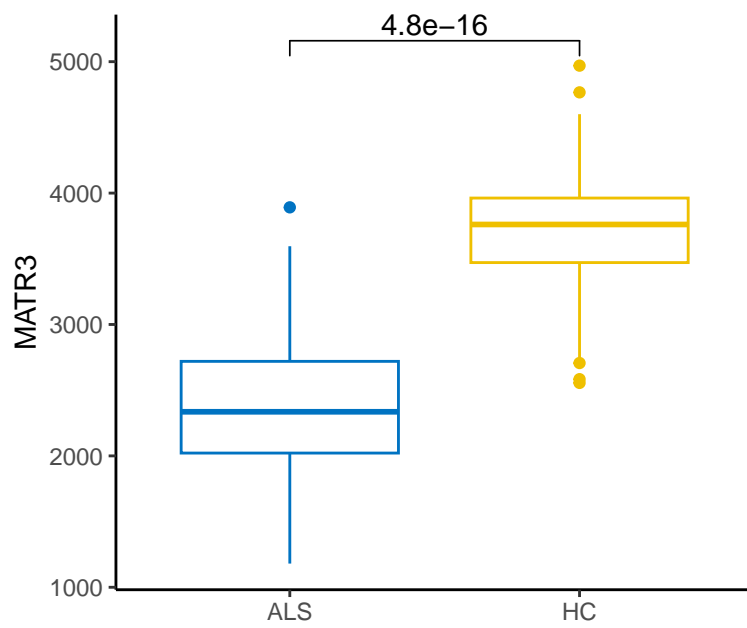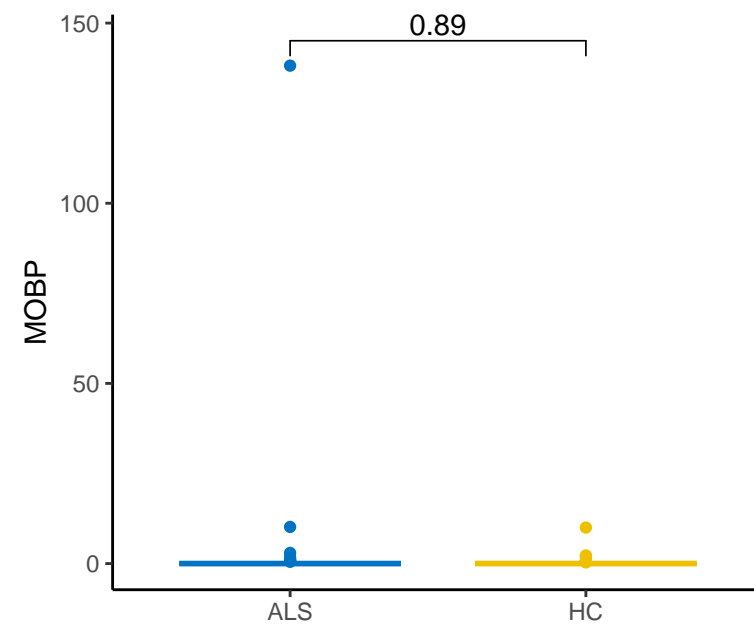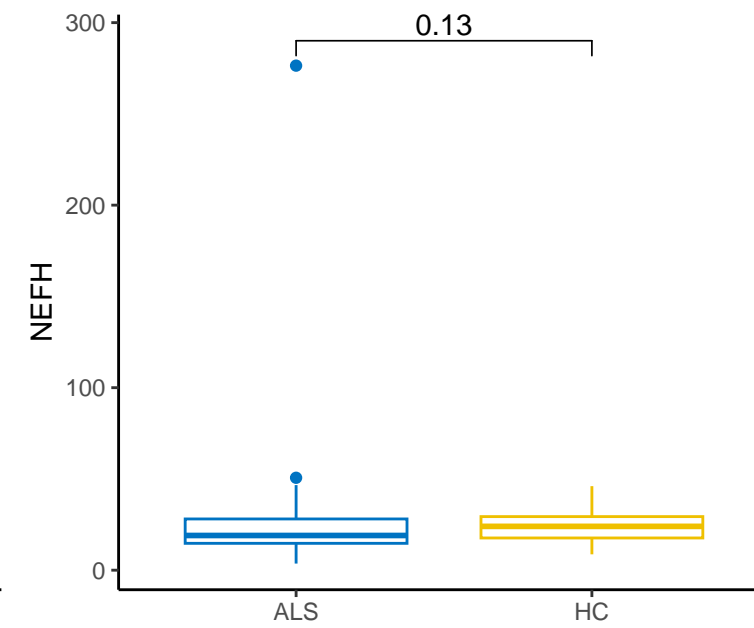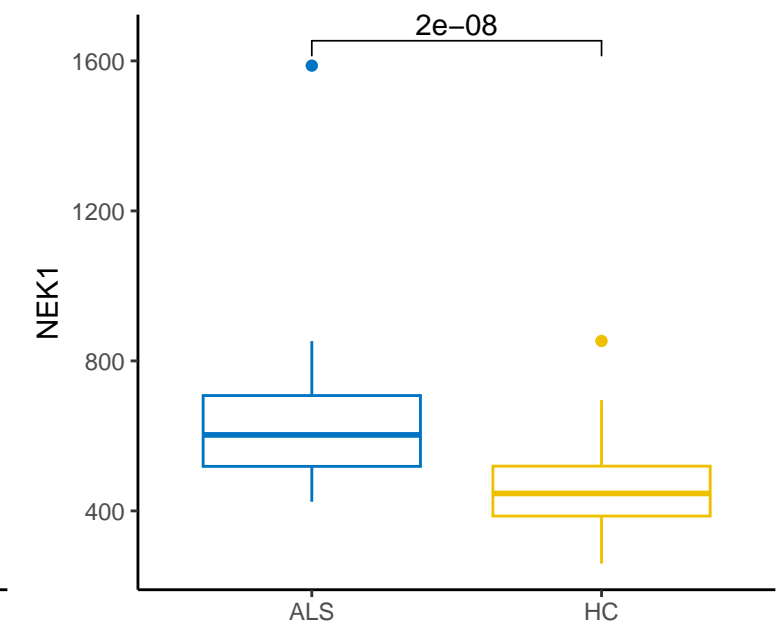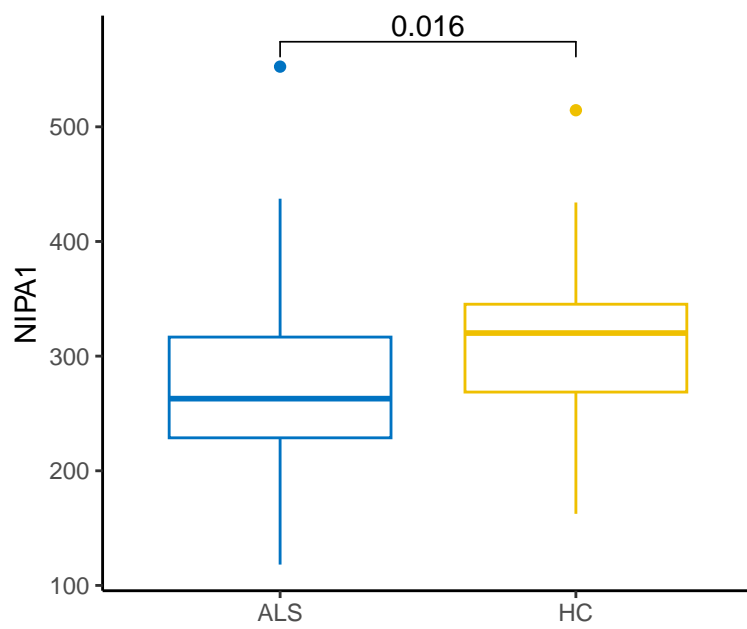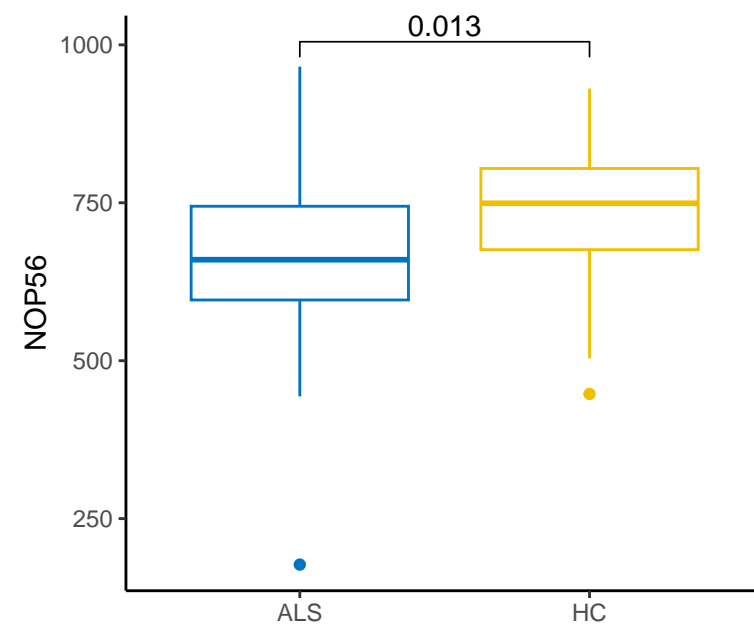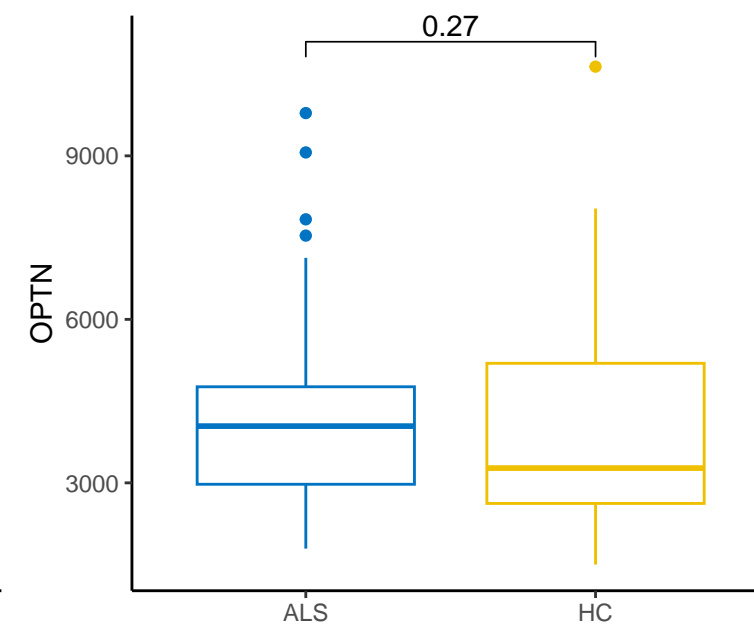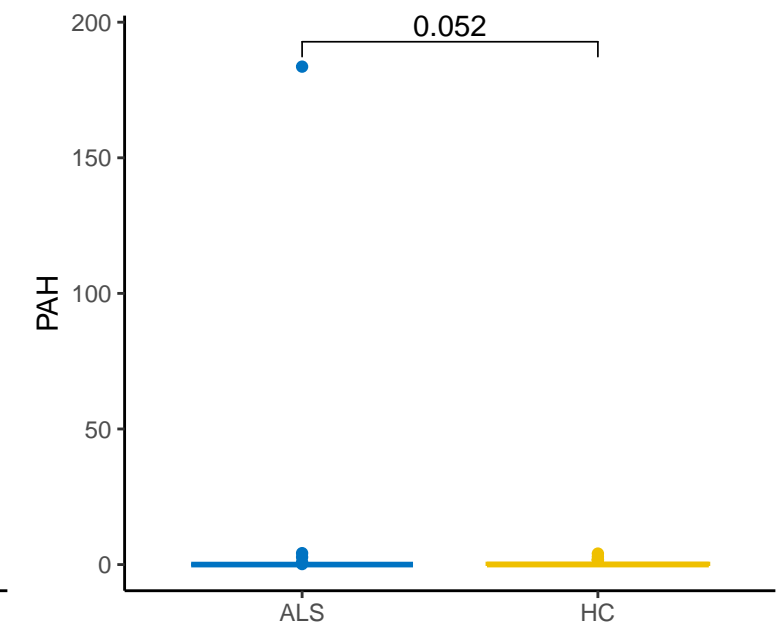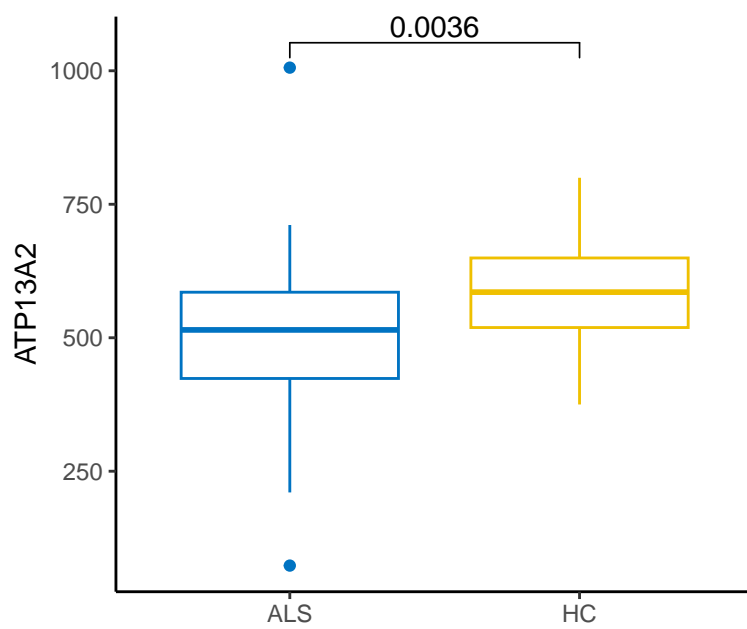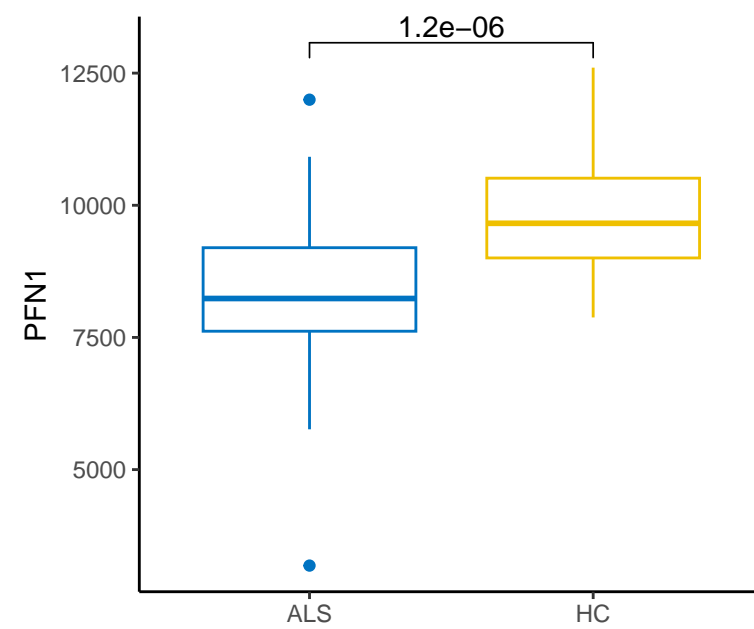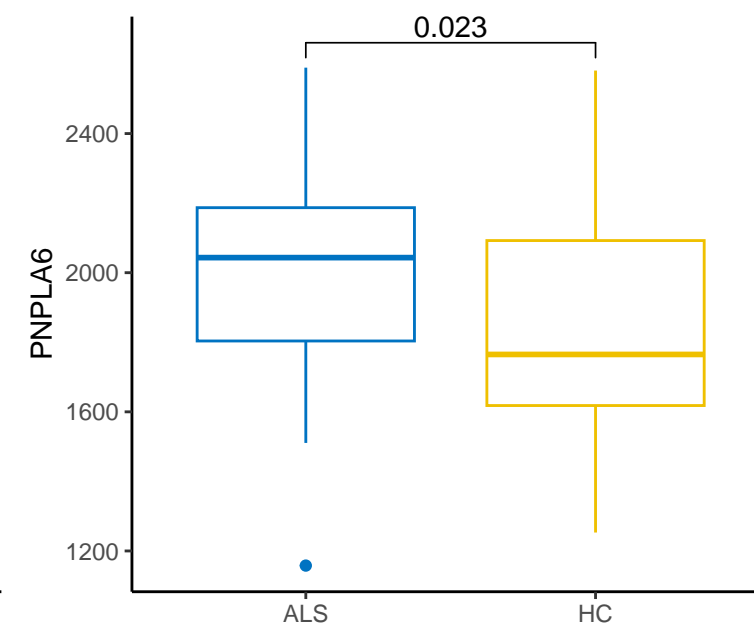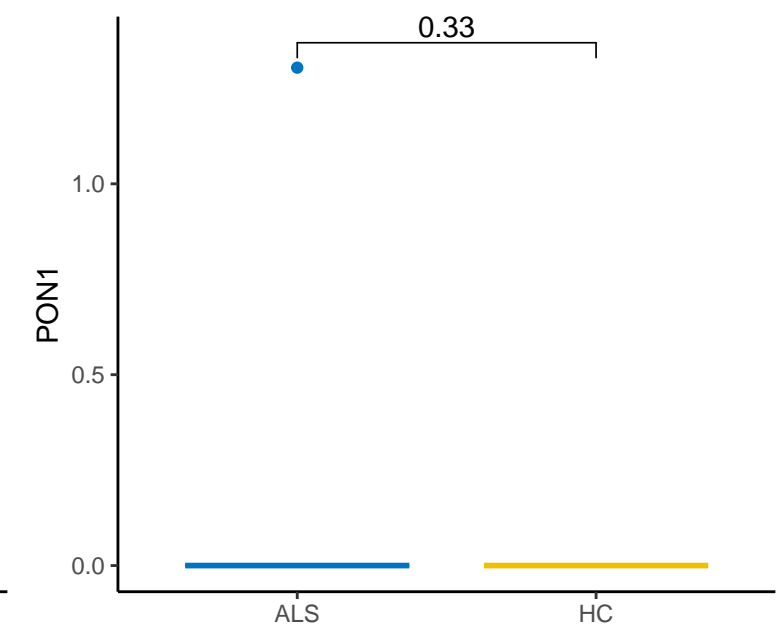

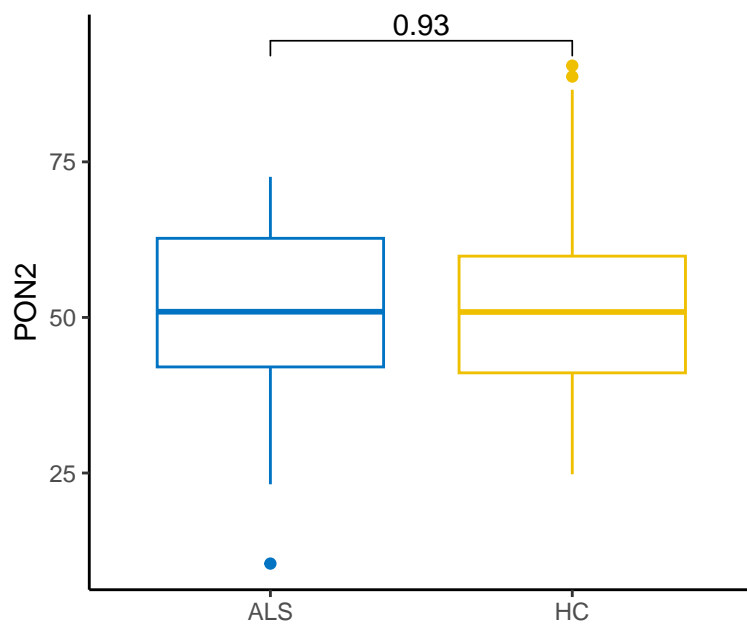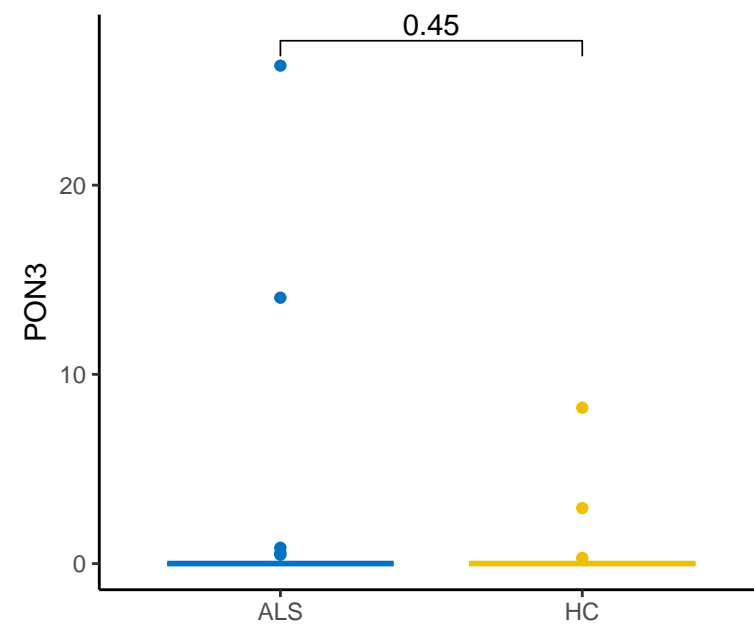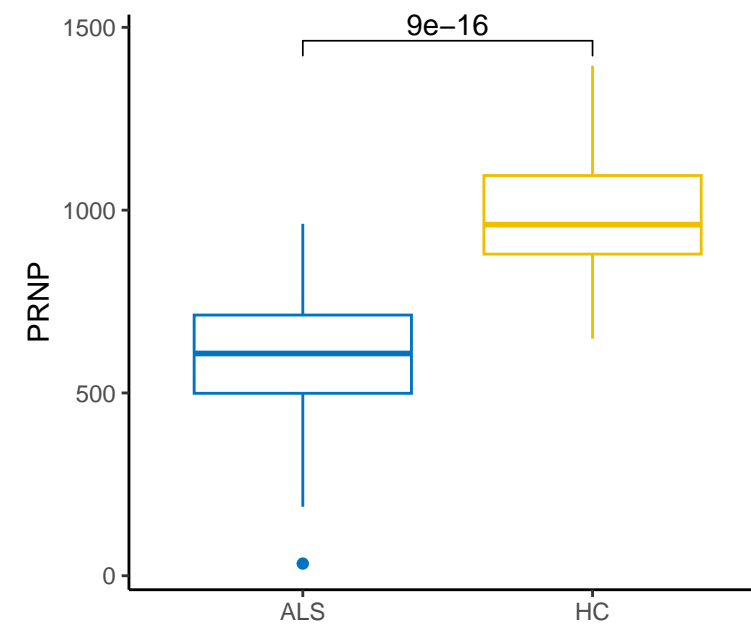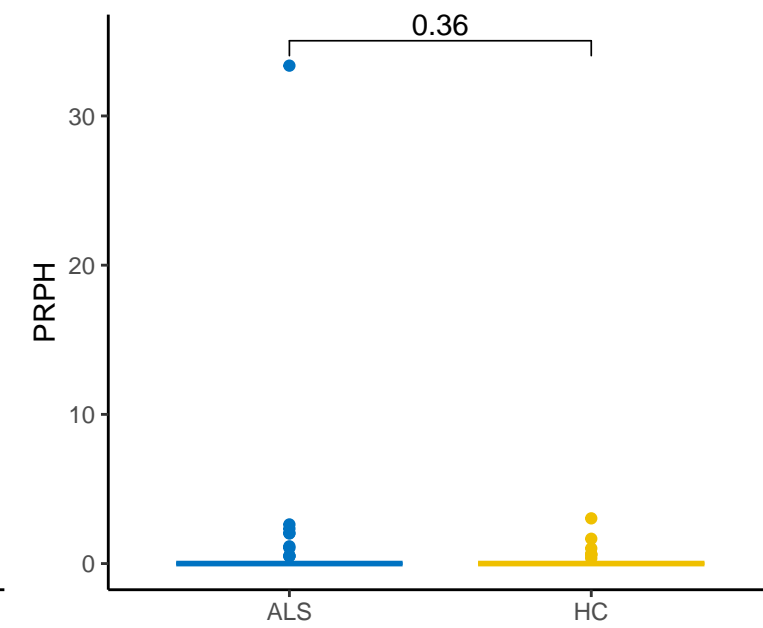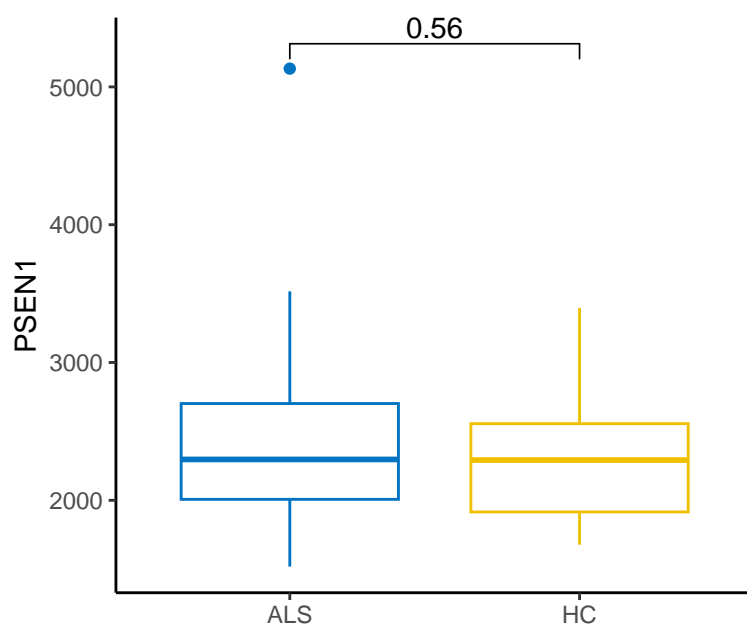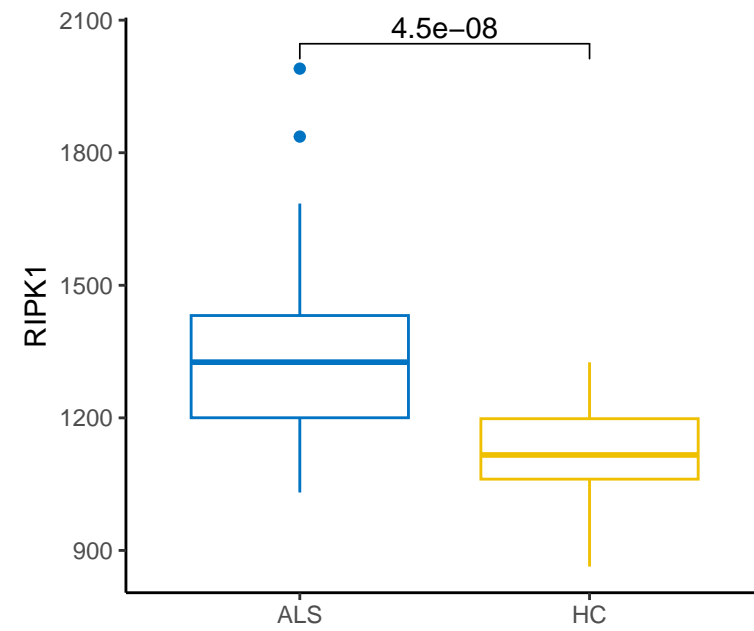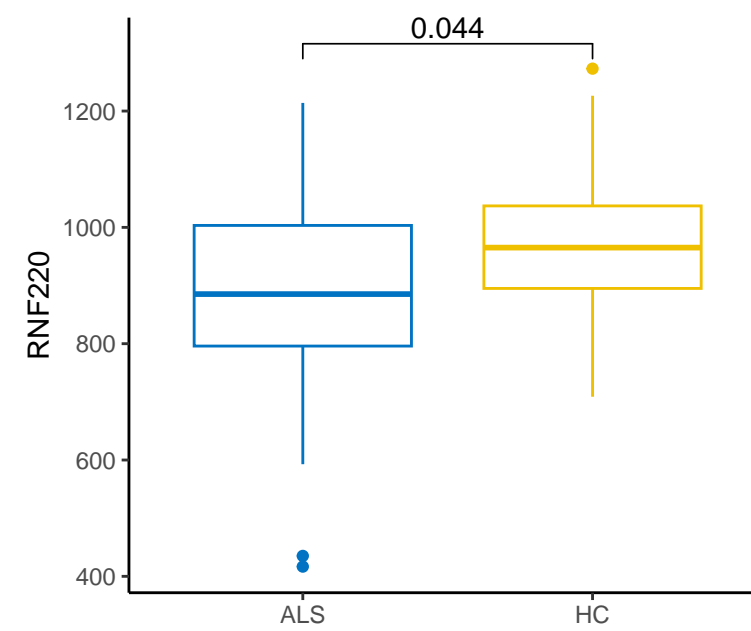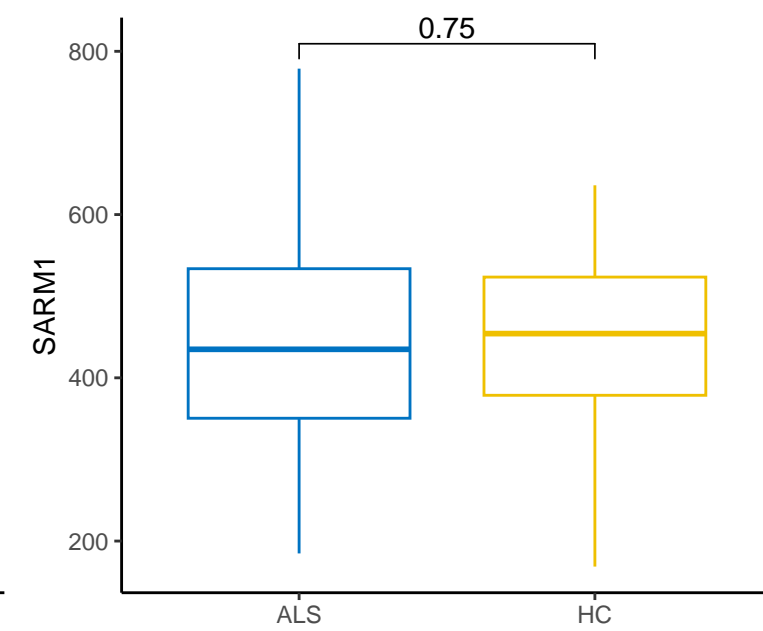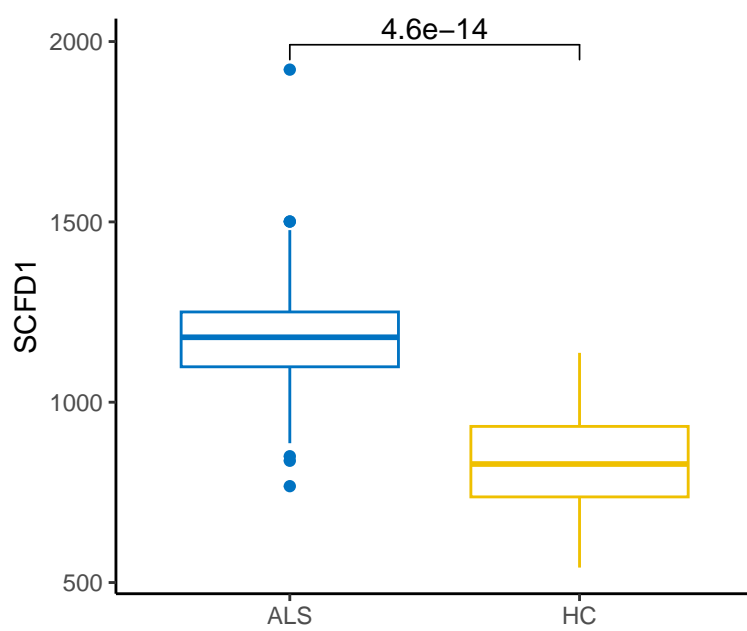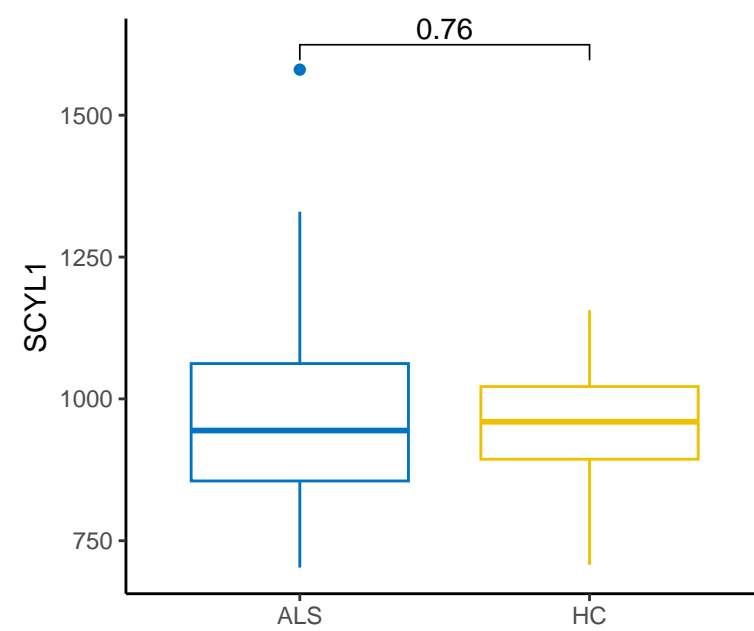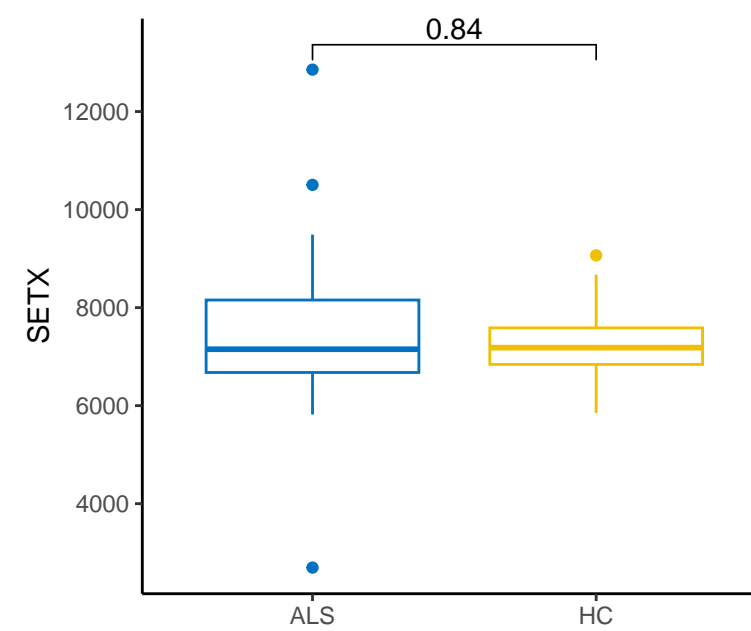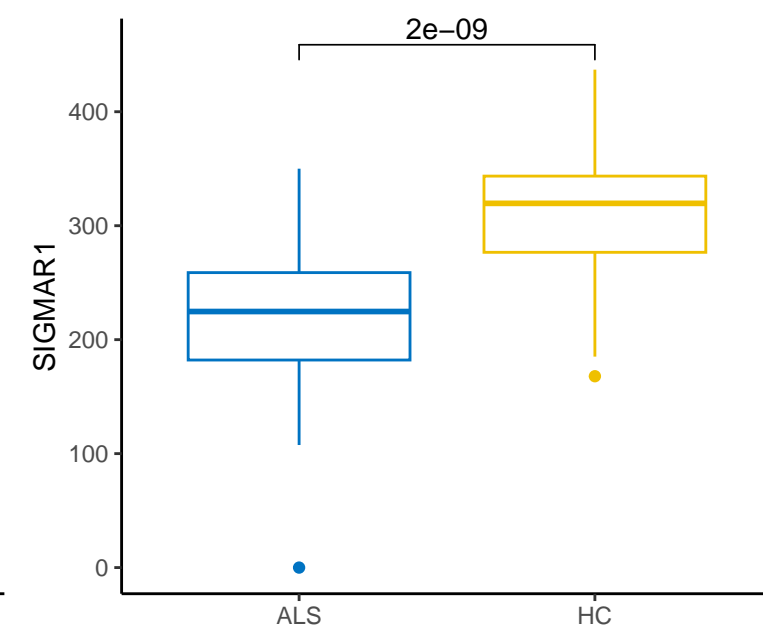

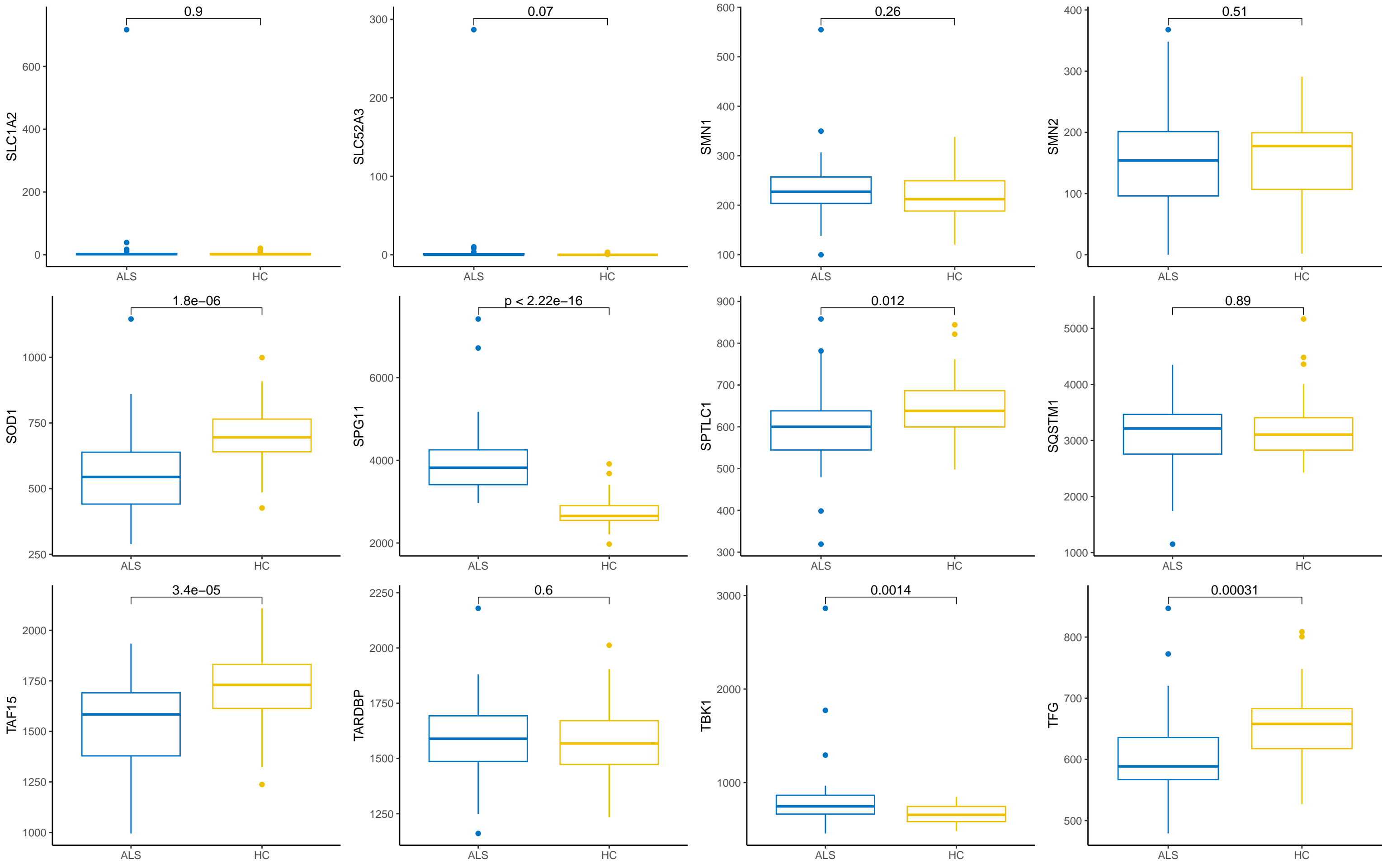

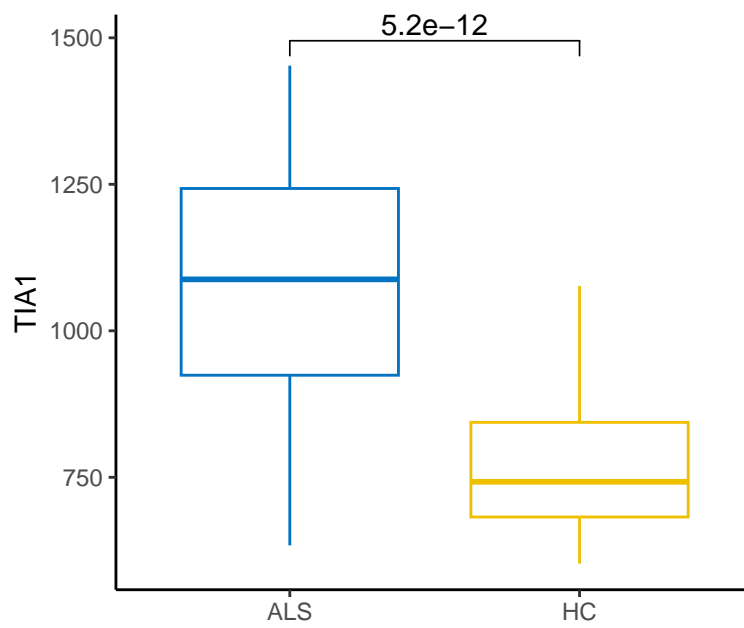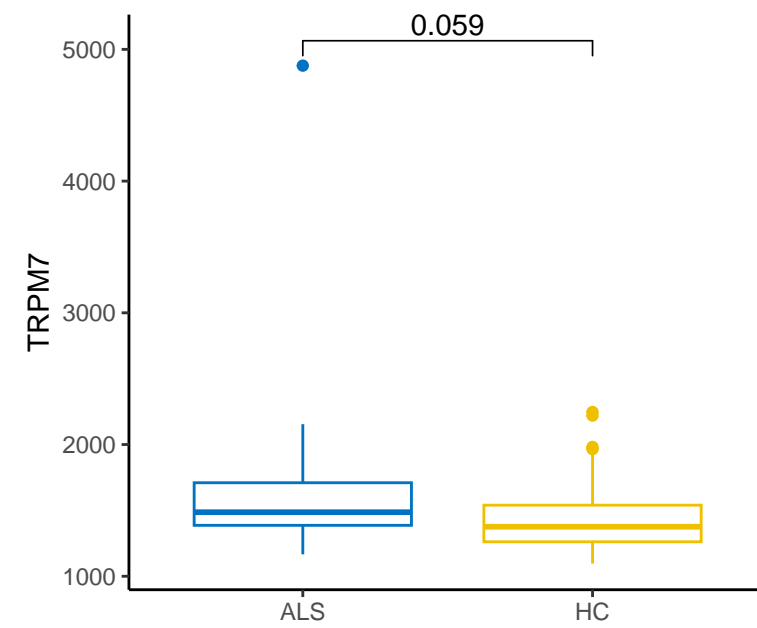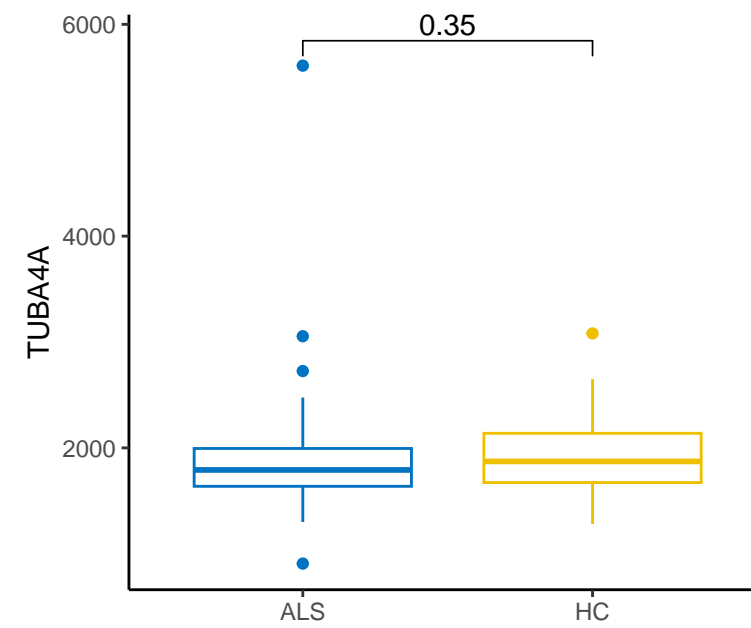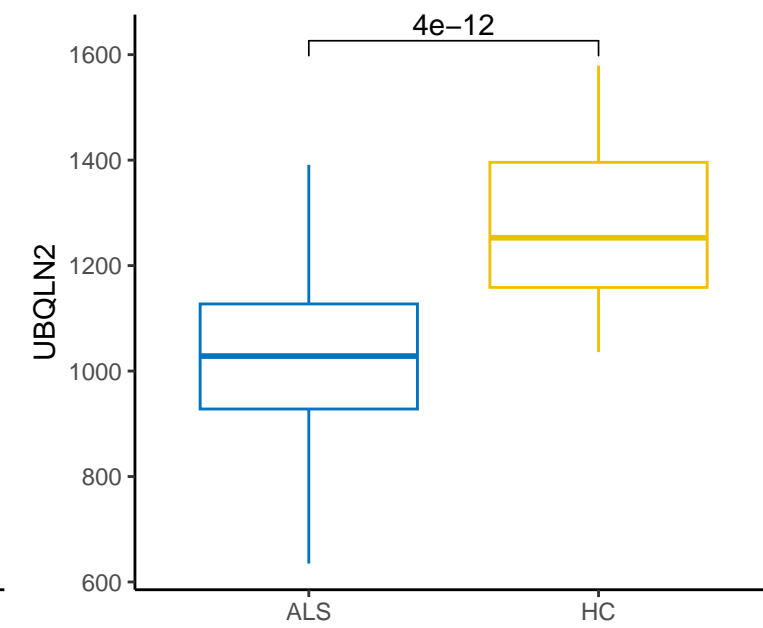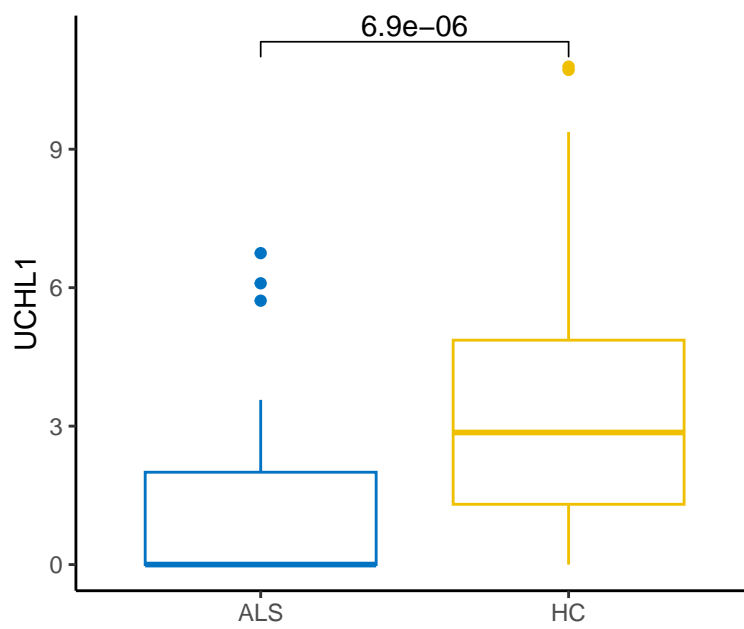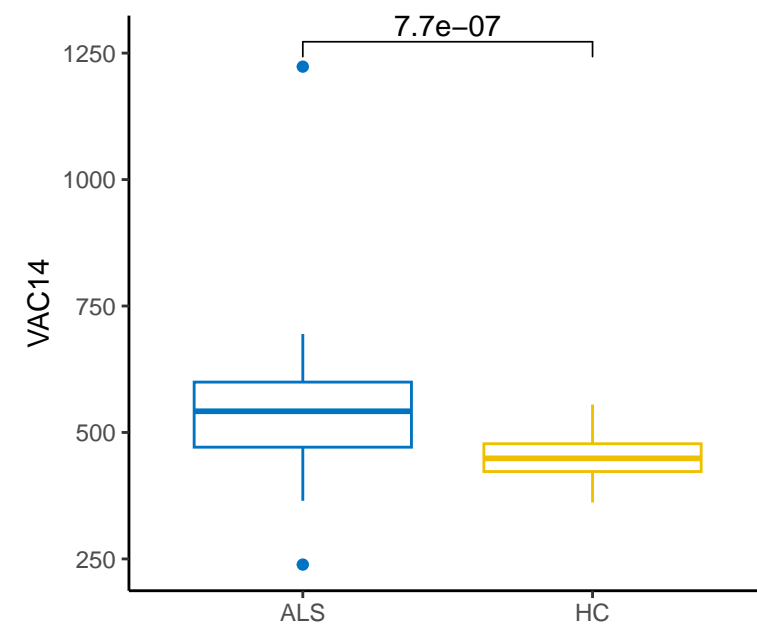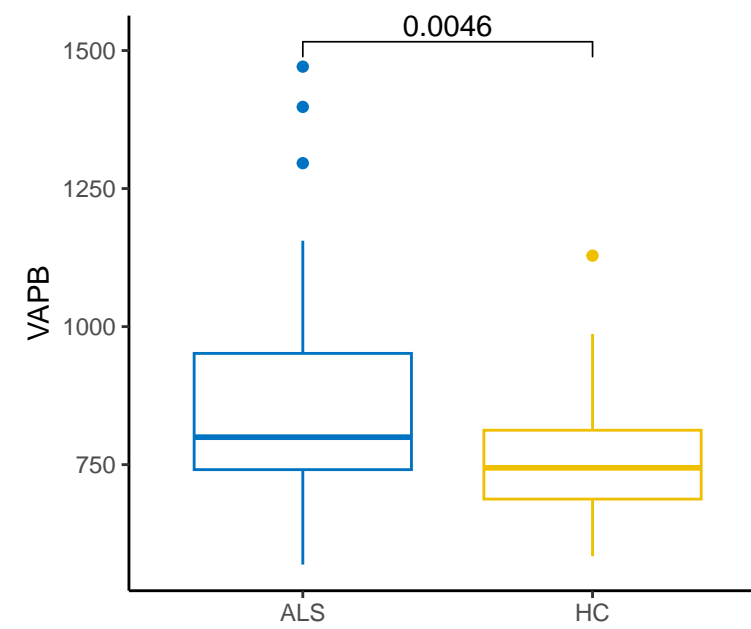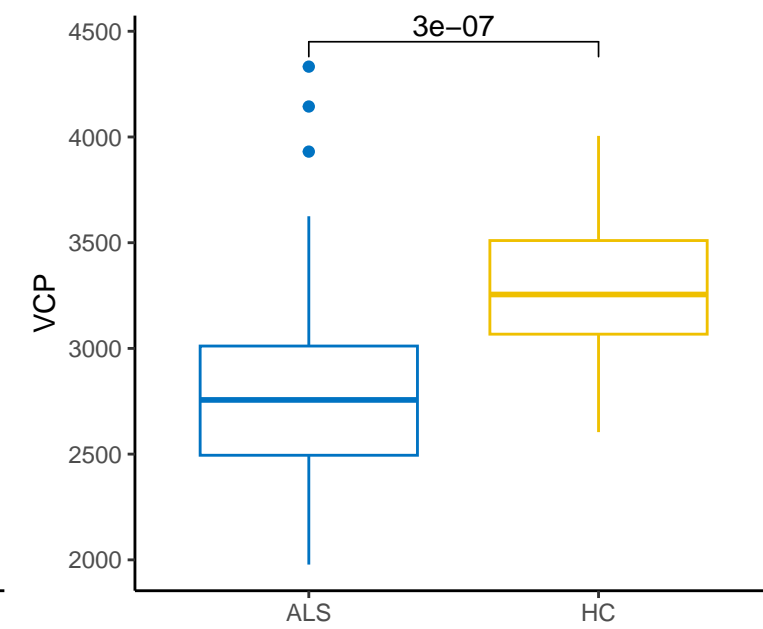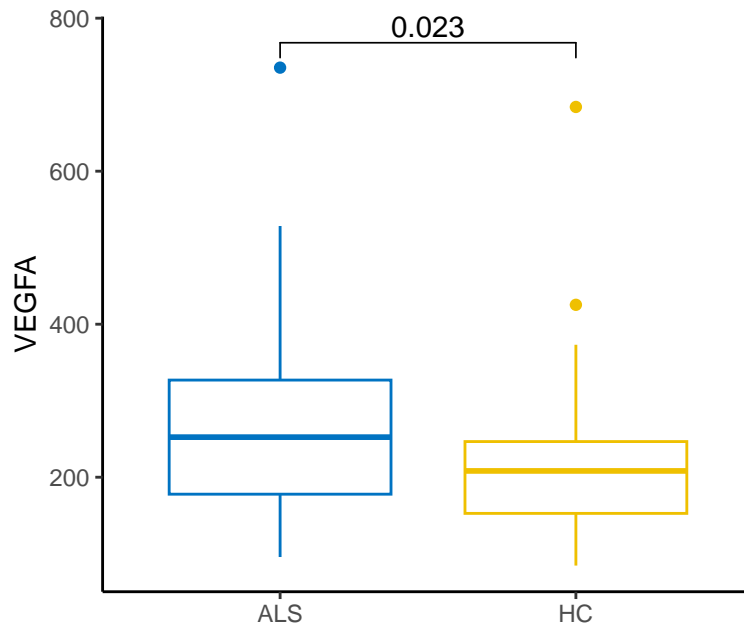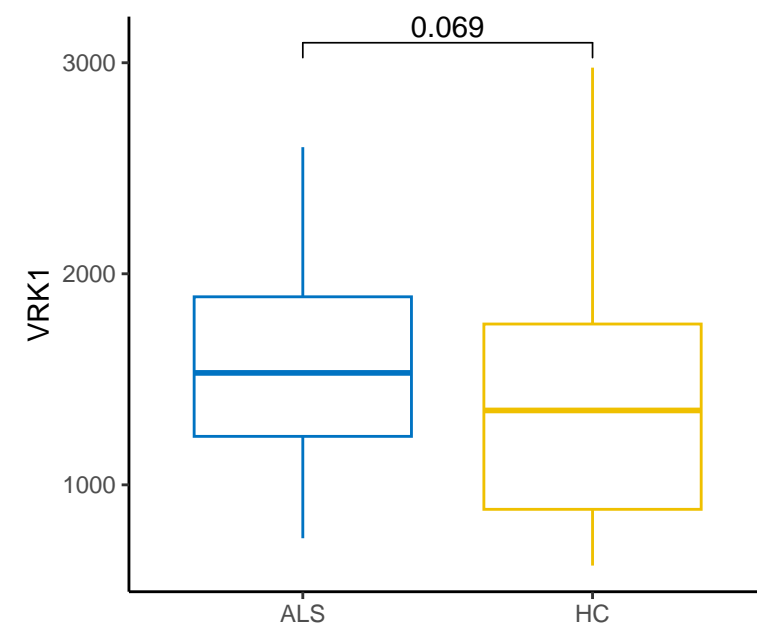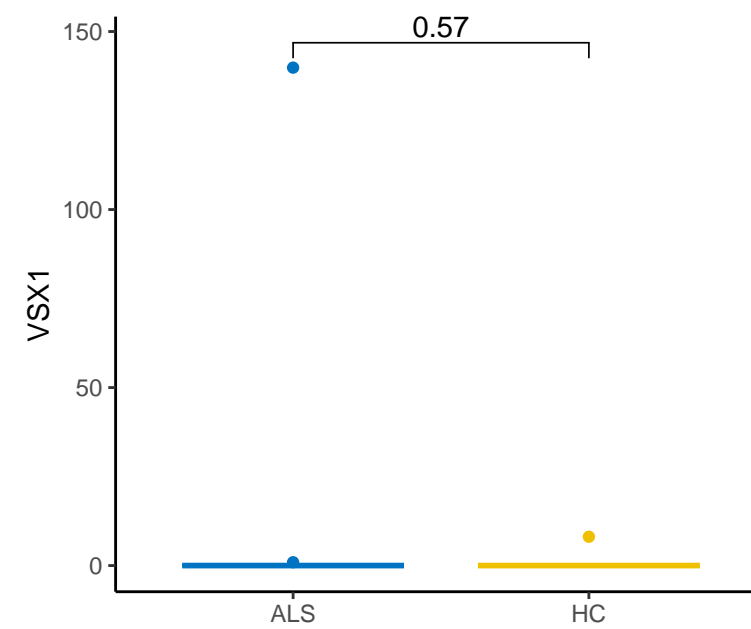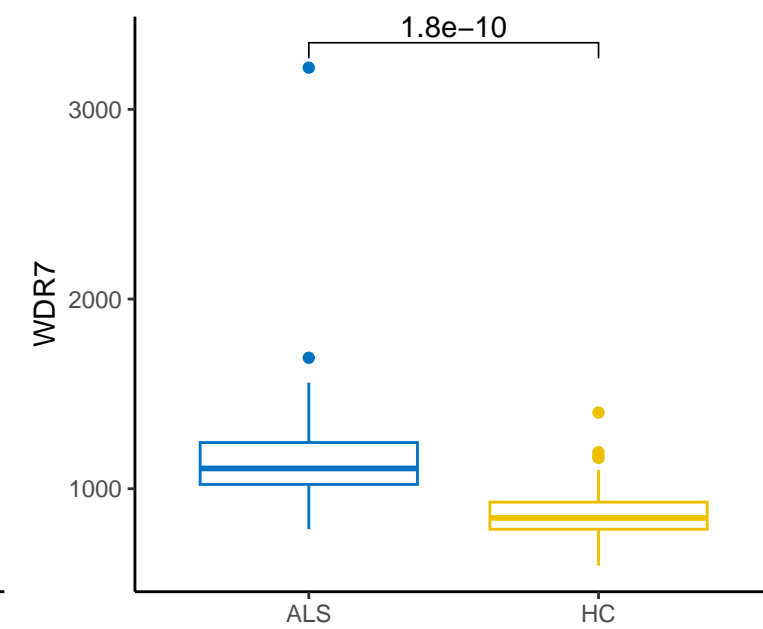

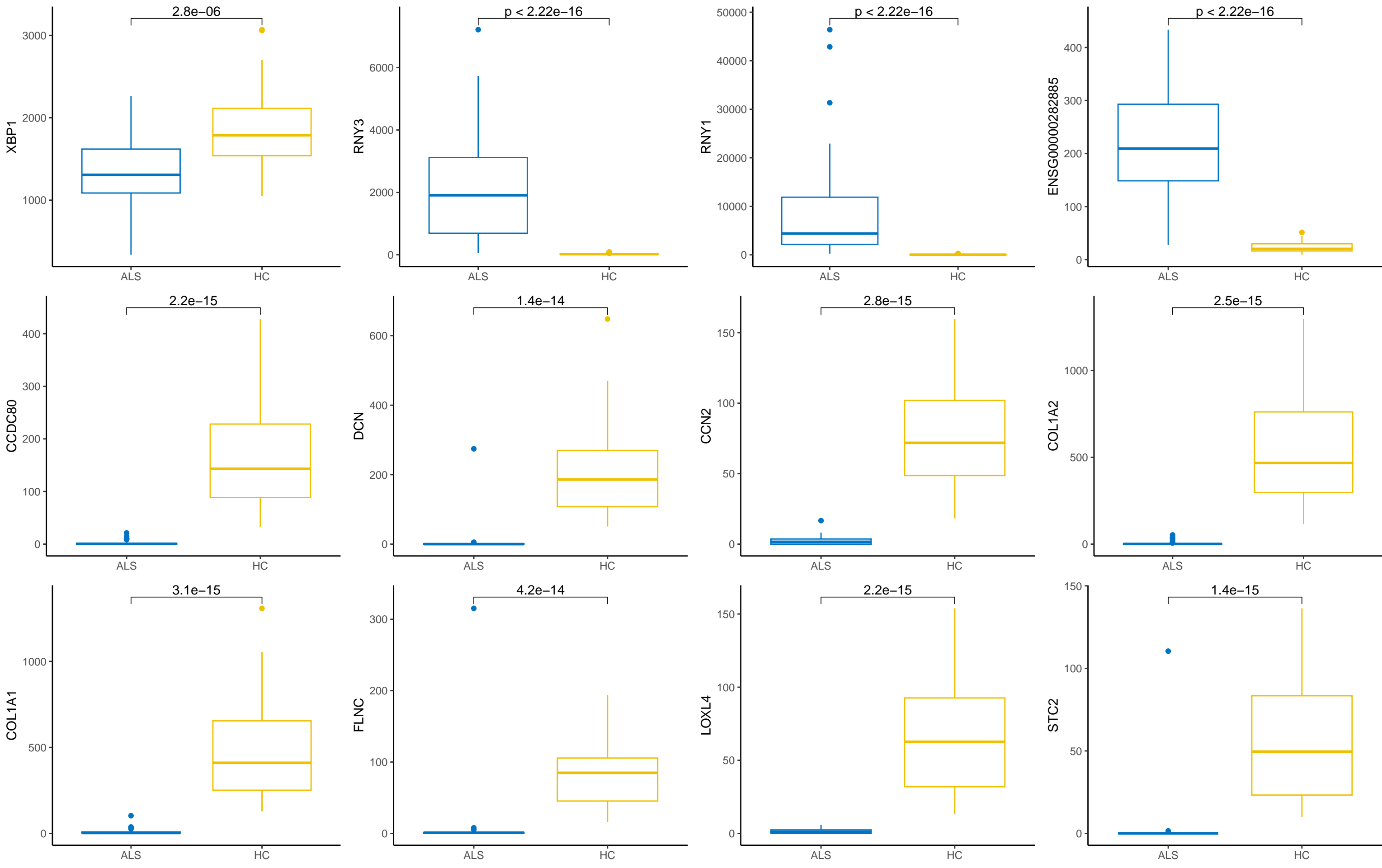

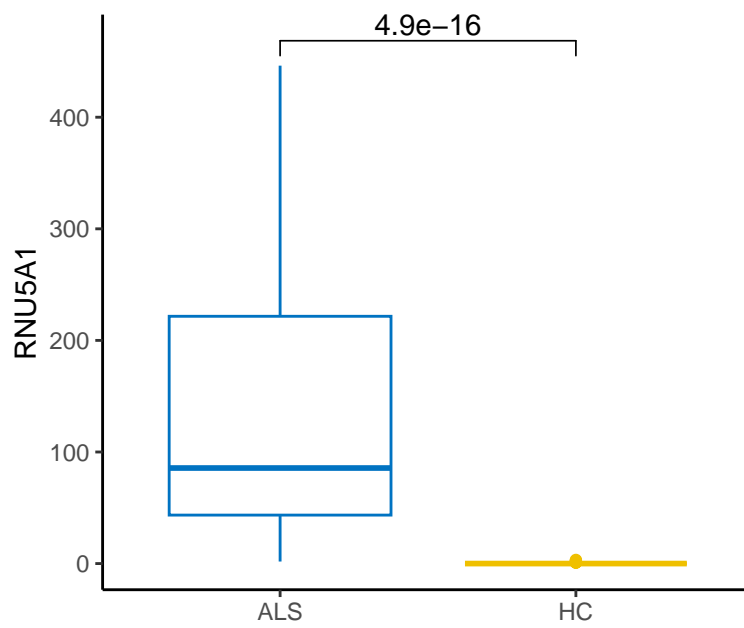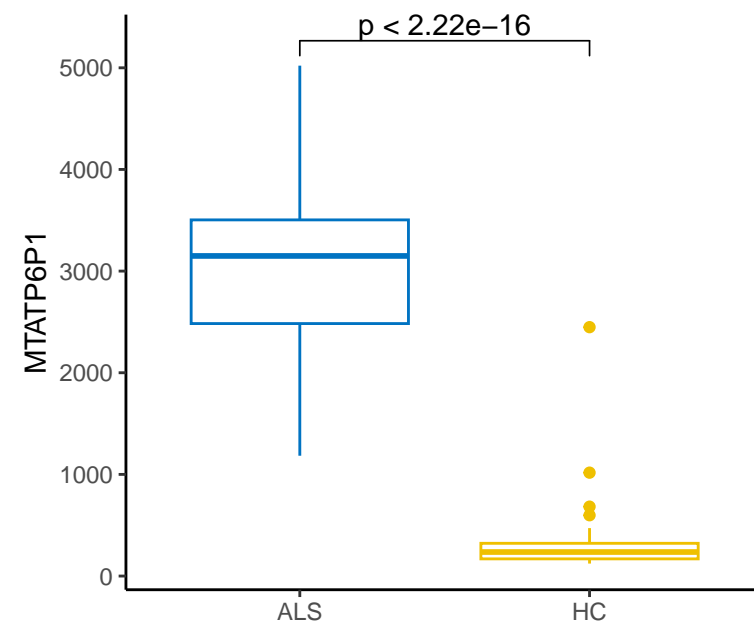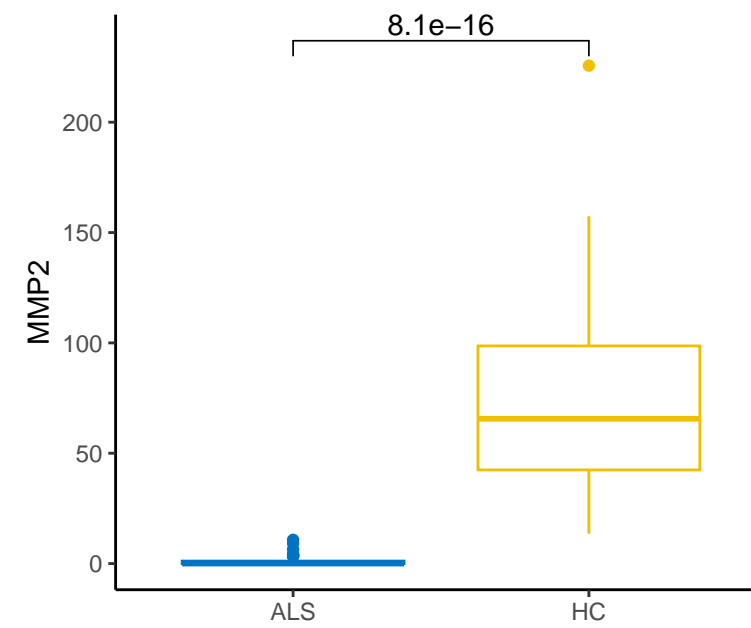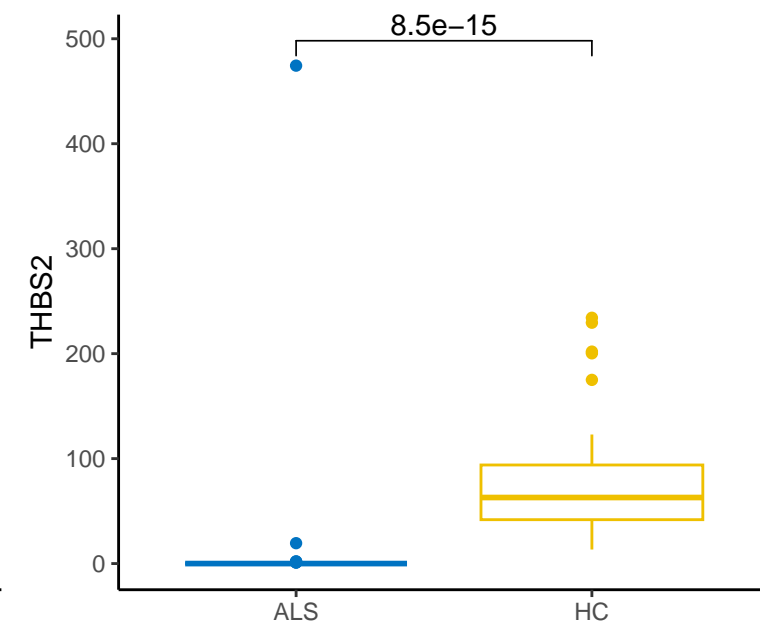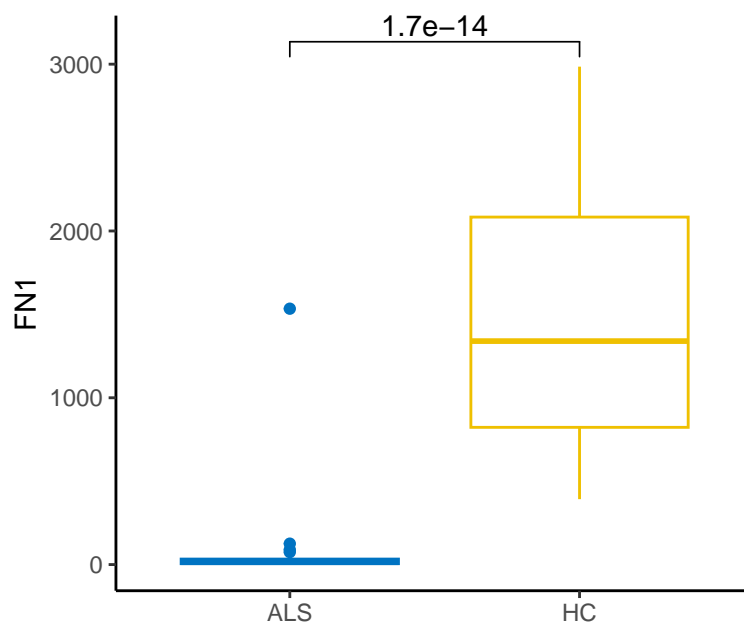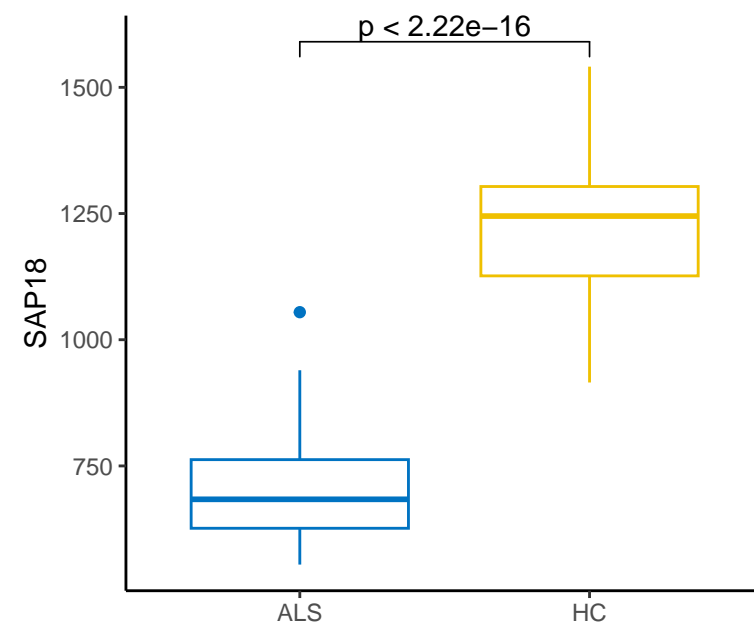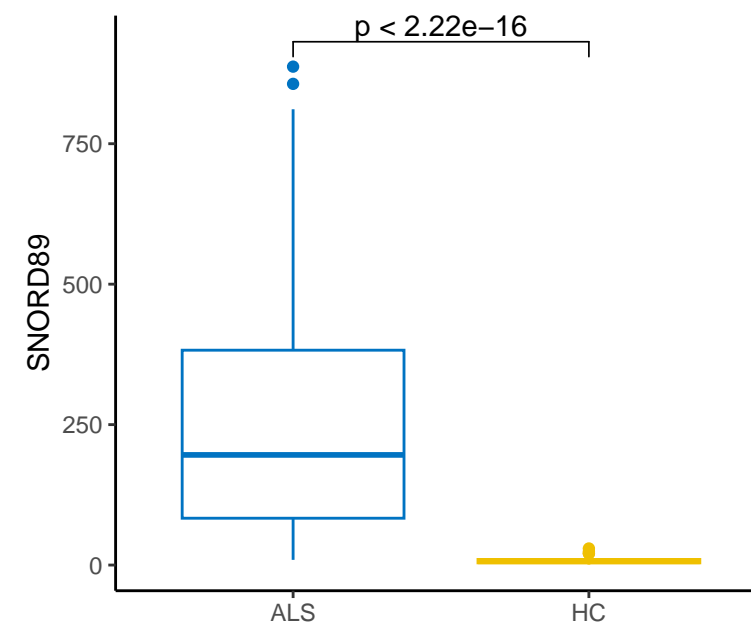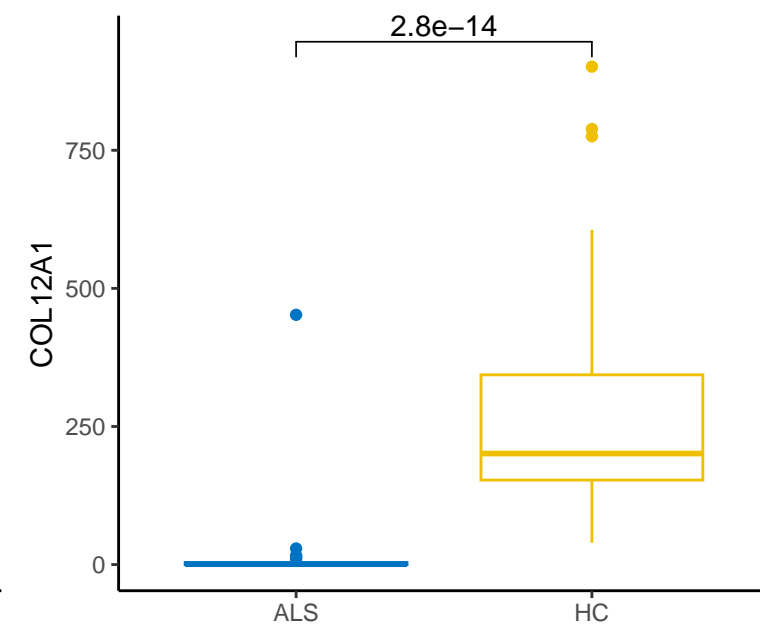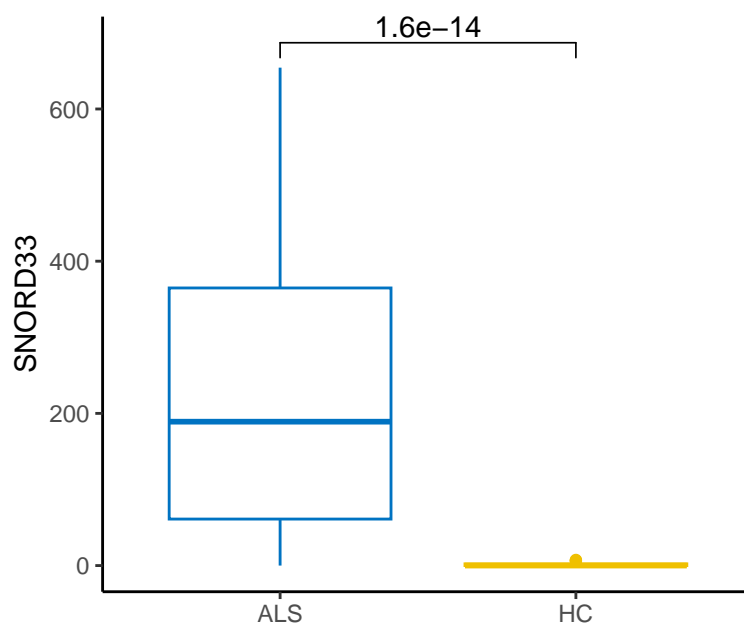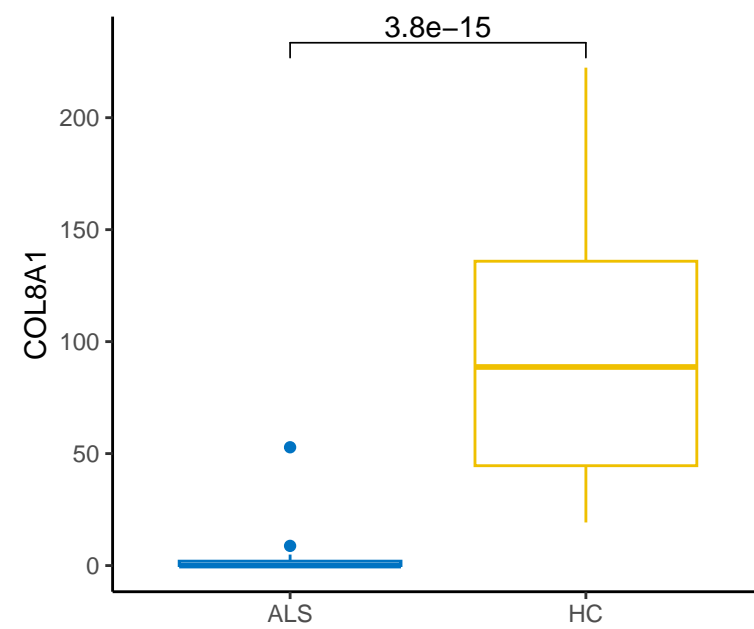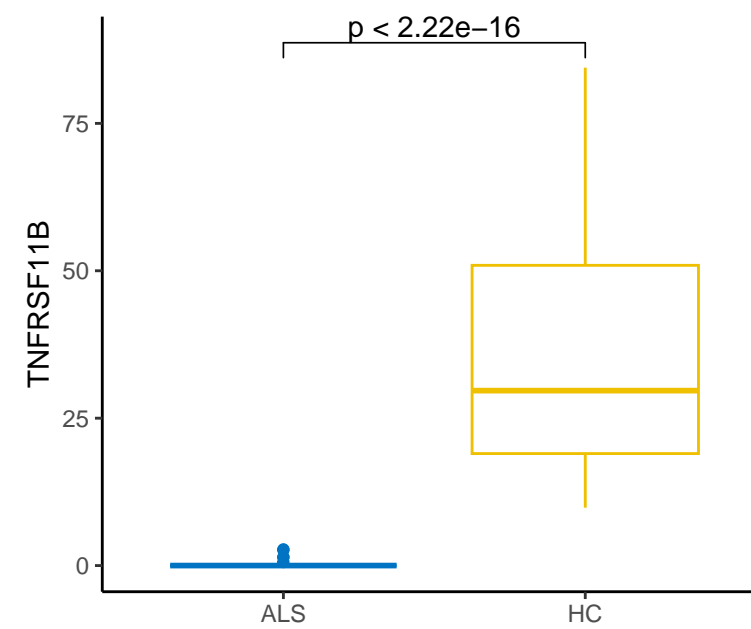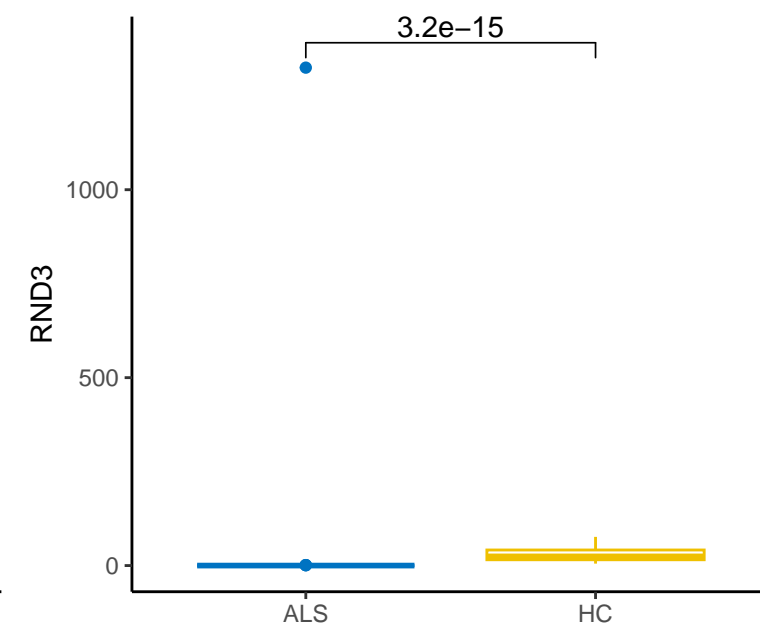

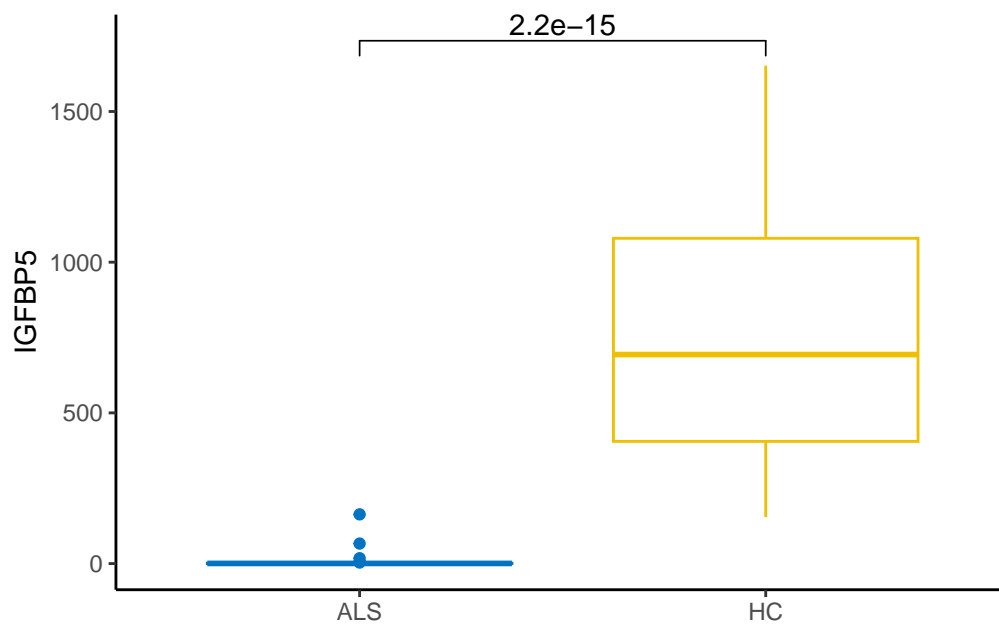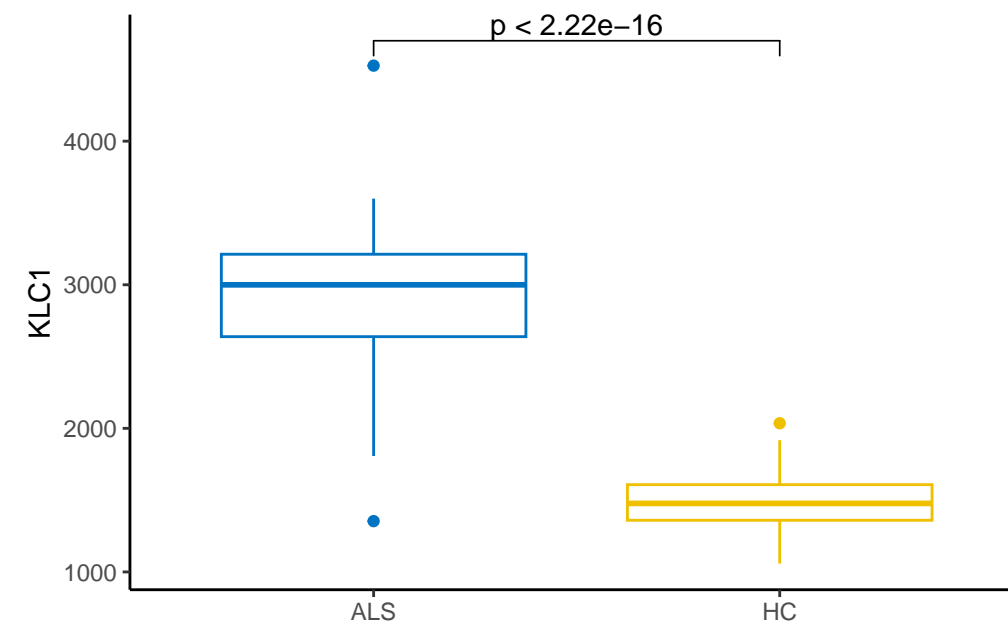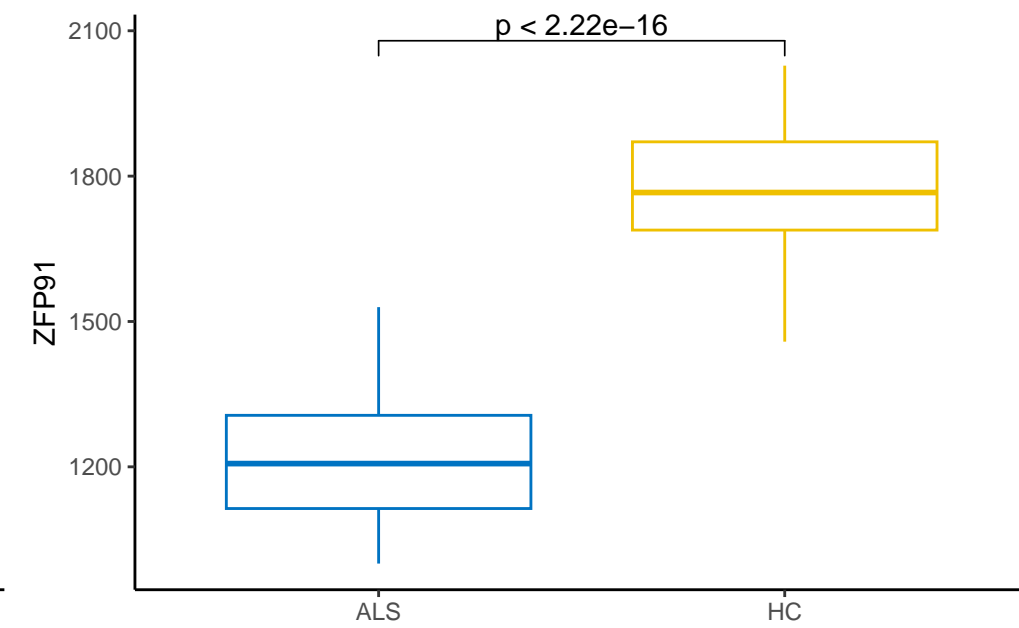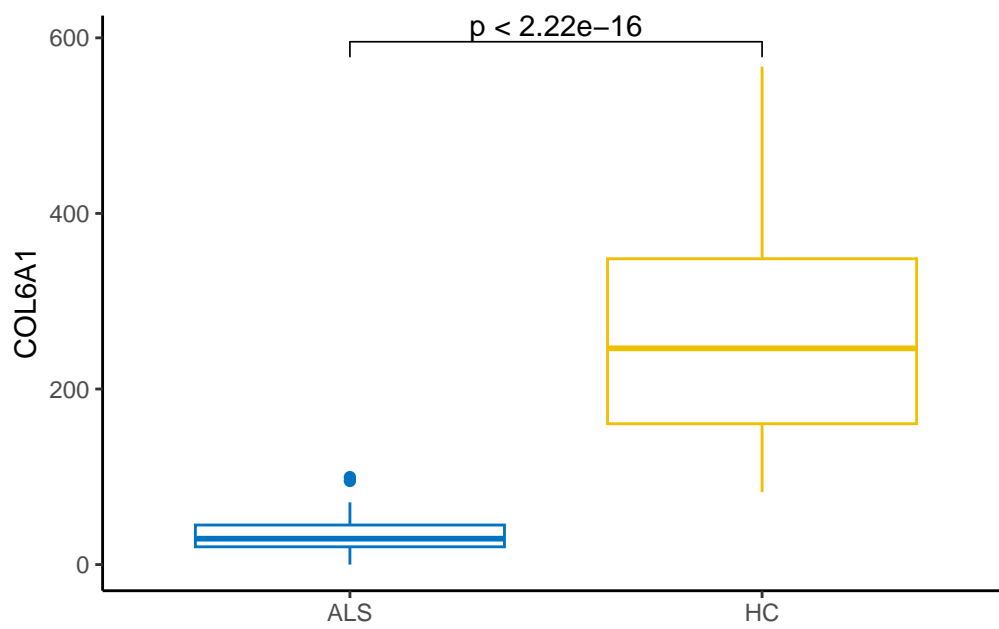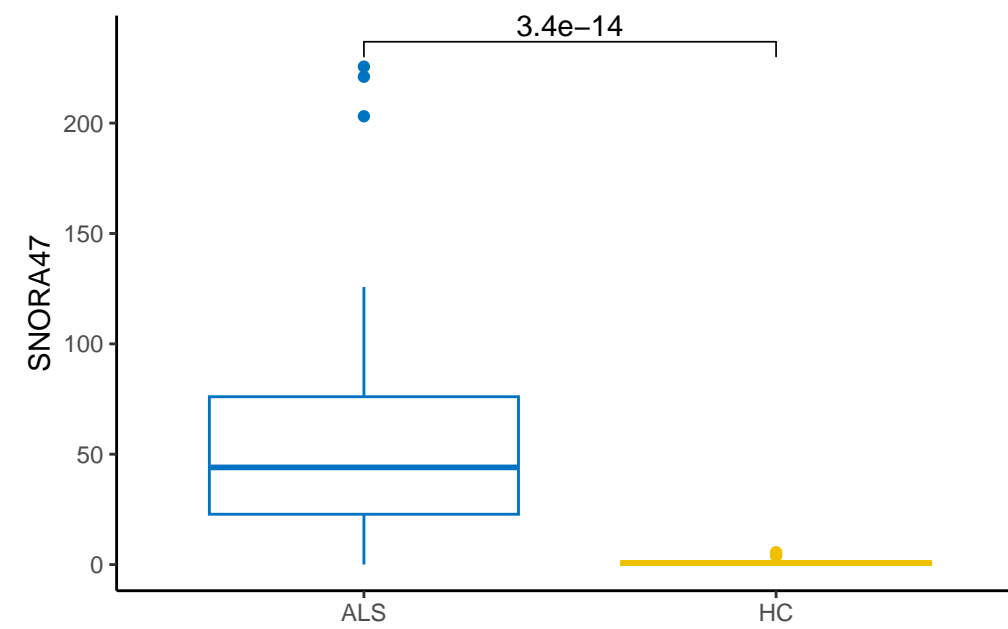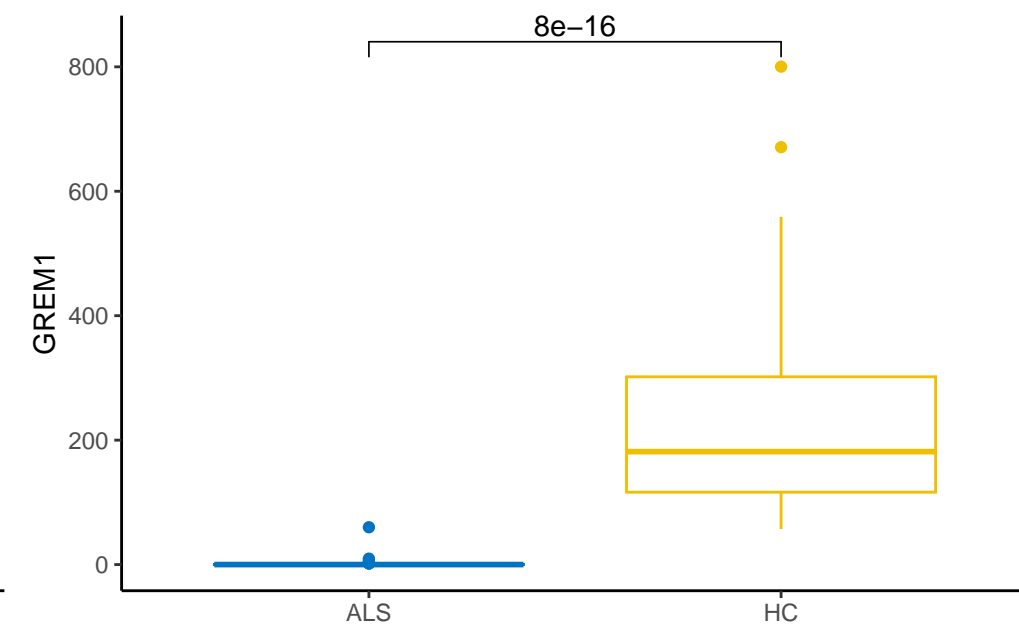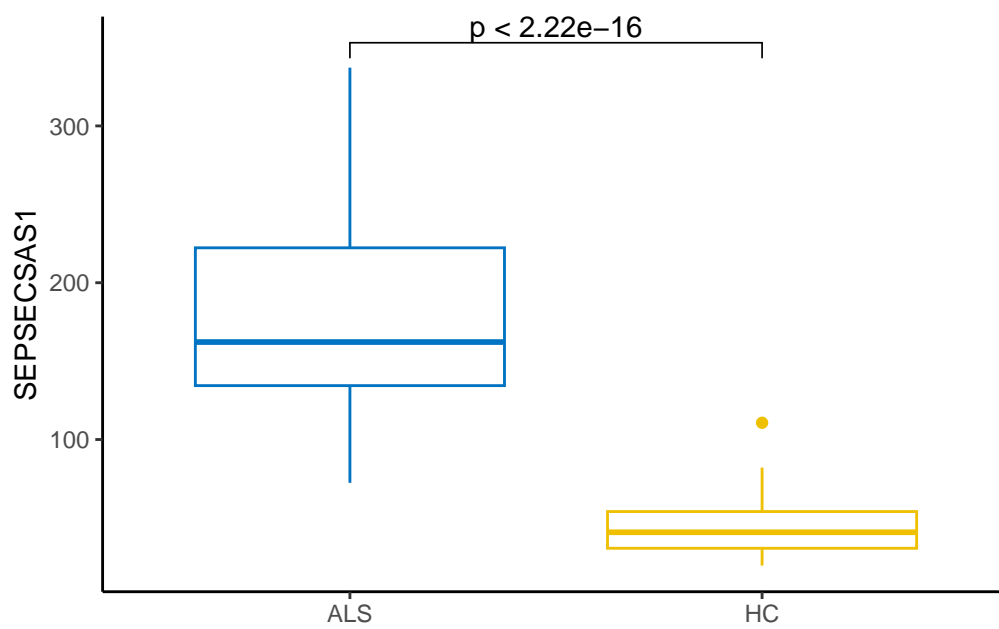

Supplement: Supplementary file 5 [file Image3.PDF]

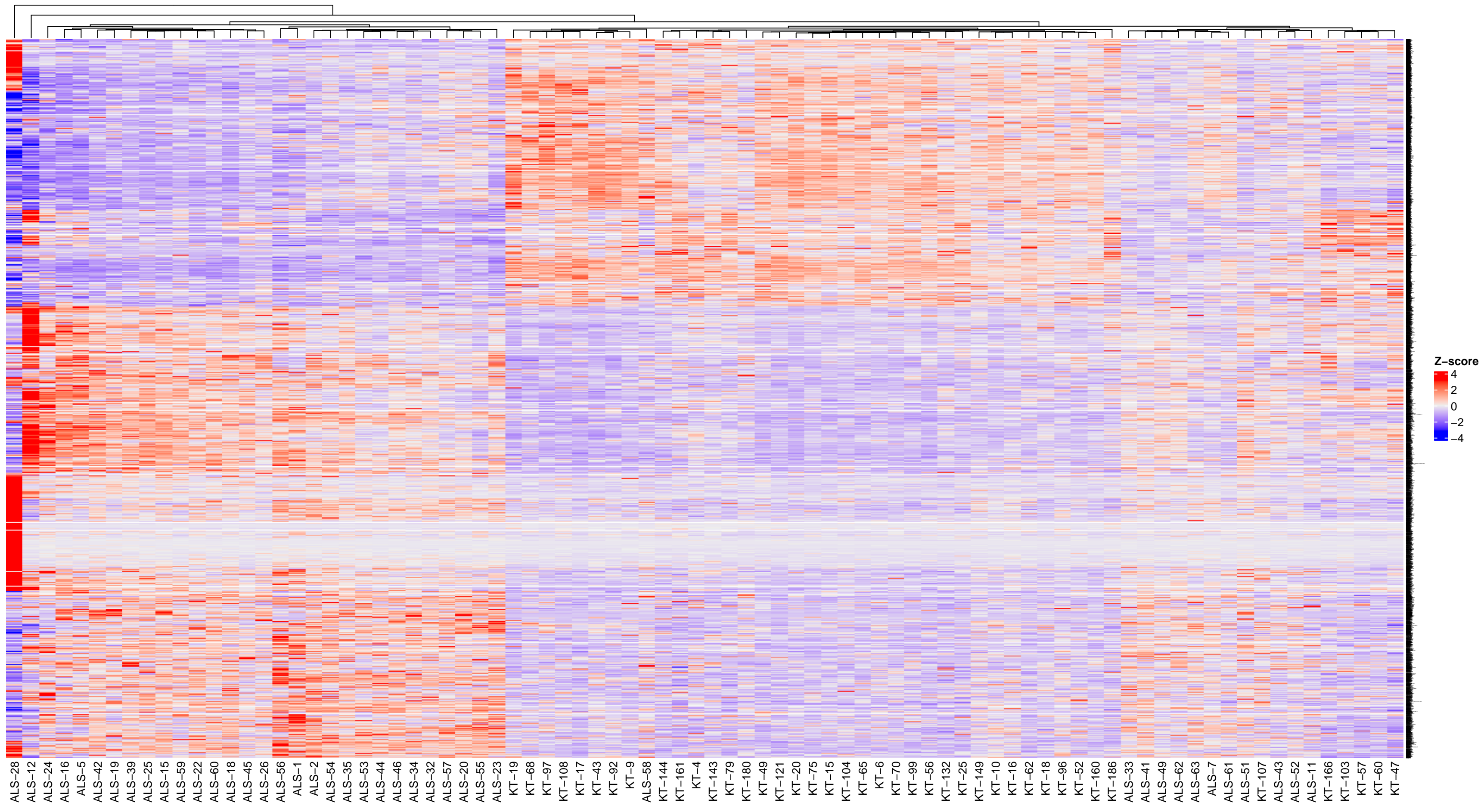

Supplement: Supplementary file 9 [file Image1.PDF]
